# ONC201 enhances the radiosensitivity of small-cell lung cancer via inhibition of the FAK signaling pathway

**DOI:** 10.1016/j.isci.2026.117202

**Published:** 2026-08-14

**Authors:** Yuheng Yan, Zhaolin Xu, Ziyang Xu, Xun Yuan, Qian Chu

**Affiliations:** 1 Department of Oncology, Tongji Hospital of Tongji Medical College, Huazhong University of Science and Technology, Wuhan 430030, P.R. China

**Keywords:** small-cell lung cancer, radiotherapy, ONC201, FAK

## Abstract

Small-cell lung cancer (SCLC) is a highly aggressive neuroendocrine malignancy with a poor prognosis. Radiotherapy is an essential treatment strategy for SCLC but is constrained by rapid relapse and therapeutic resistance. ONC201, an orally bioavailable imipridone compound, has demonstrated clinical efficacy against various malignancies. This study explored the therapeutic potential of ONC201 as a radiosensitizer in SCLC. Our results showed that ONC201 significantly suppressed SCLC cell proliferation, migration, and invasion, while promoting cell-cycle arrest and apoptosis. Moreover, ONC201 enhanced cellular radiosensitivity, augmented radiotherapy-induced G2/M phase arrest, and promoted apoptosis. Mechanistically, we confirmed that ONC201 enhanced radiosensitivity by suppressing focal adhesion kinase (FAK) signaling. Overall, our findings underscore the potential of ONC201 as a promising radiosensitizer for SCLC treatment.

## Highlights

• ONC201 enhances the radiosensitivity of SCLC cells
• ONC201 potentiates radiation-induced cell-cycle arrest and apoptosis
• ONC201-mediated radiosensitization is associated with suppression of FAK signaling
• The combination of ONC201 and radiotherapy suppresses SCLC tumor growth *in vivo*

## Graphical abstract

**Figure:**
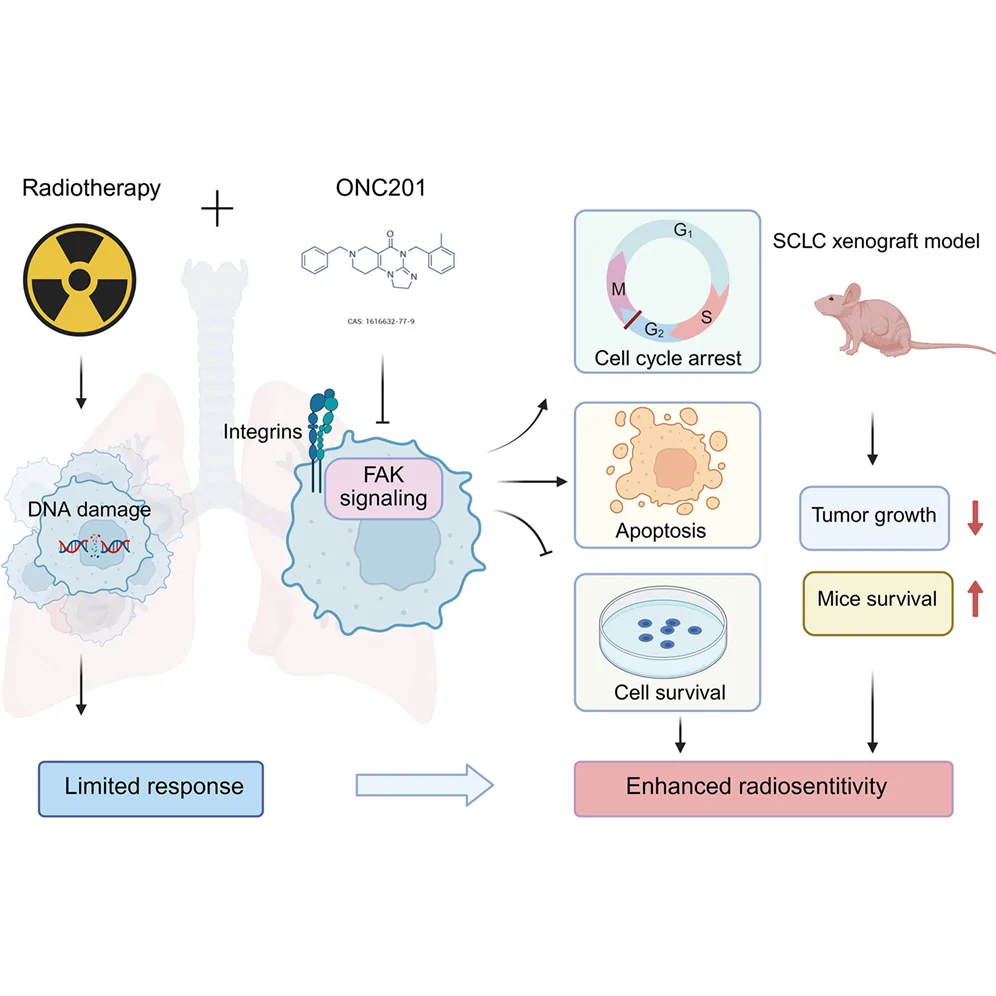

## Teaser

Therapy; Molecular network; Cancer

## Introduction

Lung cancer has the highest incidence and mortality rates worldwide.^1^^,^^2^ Small-cell lung cancer (SCLC), a highly aggressive neuroendocrine carcinoma, accounts for approximately 15% of all lung cancer cases.^3^ Approximately 70% of SCLC patients are diagnosed at an advanced stage with distant metastases, conferring an extremely poor prognosis.^4^ Radiotherapy plays a critical role in SCLC treatment. Patients with limited-stage SCLC achieve a 2-year overall survival (OS) rate exceeding 50% when treated with curative thoracic chemoradiotherapy.^5^ For patients with extensive-stage SCLC, consolidative thoracic radiotherapy following standard chemotherapy significantly improves the 2-year OS rate (13% vs. 3%, *p* = 0.004).^6^ Furthermore, radiotherapy effectively alleviates local symptoms (e.g., pain and dyspnea), thereby enhancing the quality of life. However, the efficacy of radiotherapy alone is limited in achieving systemic disease control, and patients remain at substantial risk of recurrence and distant metastasis. Consequently, investigating combination strategies that integrate radiotherapy with systemic therapies (e.g., targeted therapy and immunotherapy) is a major focus of current research.^7^^,^^8^

ONC201 is an imipridone compound that has attracted much attention in recent years. Initially identified as a tumor necrosis factor-related apoptosis-inducing ligand (TRAIL)-inducing compound 10 (TIC10), ONC201 promotes the nuclear translocation of the transcription factor forkhead box protein O3a (FoxO3a), thereby inducing p53-independent TRAIL expression and activating the caspase-8-dependent extrinsic apoptotic pathway.^9^ ONC201 exhibits favorable oral bioavailability, safety profile, and blood-brain barrier penetration, demonstrating its clinical potential in various cancers.^10^^,^^11^^,^^12^^,^^13^^,^^14^ Multiple preclinical studies have reported ONC201’s role as a radiosensitizer. For instance, ONC201 significantly sensitized prostate cancer cells to radiation by suppressing cell-cycle progression and DNA damage repair (DDR).^15^ Similarly, He et al. confirmed that ONC201 enhances radiosensitivity in glioblastoma by inhibiting glioma stem cell proliferation and regulating the cell cycle.^16^ It was also reported that ONC201 treatment increased the levels of DNA breakage-related proteins such as phosphorylated Chk1, Wee1, and γ-H2AX in SCLC cells,^17^ although whether ONC201 acts as a radiosensitizer in SCLC remains unknown.

Given the limited therapeutic options for SCLC and the need to improve radiotherapy efficacy, we investigated whether ONC201 could enhance the radiosensitivity of SCLC and explored the underlying mechanisms. Using multiple molecularly distinct SCLC cell lines and xenograft models, we demonstrate that ONC201 suppresses SCLC cell growth and potentiates the antitumor effects of radiotherapy both *in vitro* and *in vivo*. Mechanistically, bulk RNA sequencing (RNA-seq) analysis and functional validation indicate that ONC201-mediated radiosensitization is associated with suppression of focal adhesion kinase (FAK) signaling. These findings provide preclinical evidence supporting ONC201 as a potential radiosensitizer for SCLC.

## Results

### ONC201 inhibits the proliferation, migration, and invasion of SCLC cells

**Figure 1:**
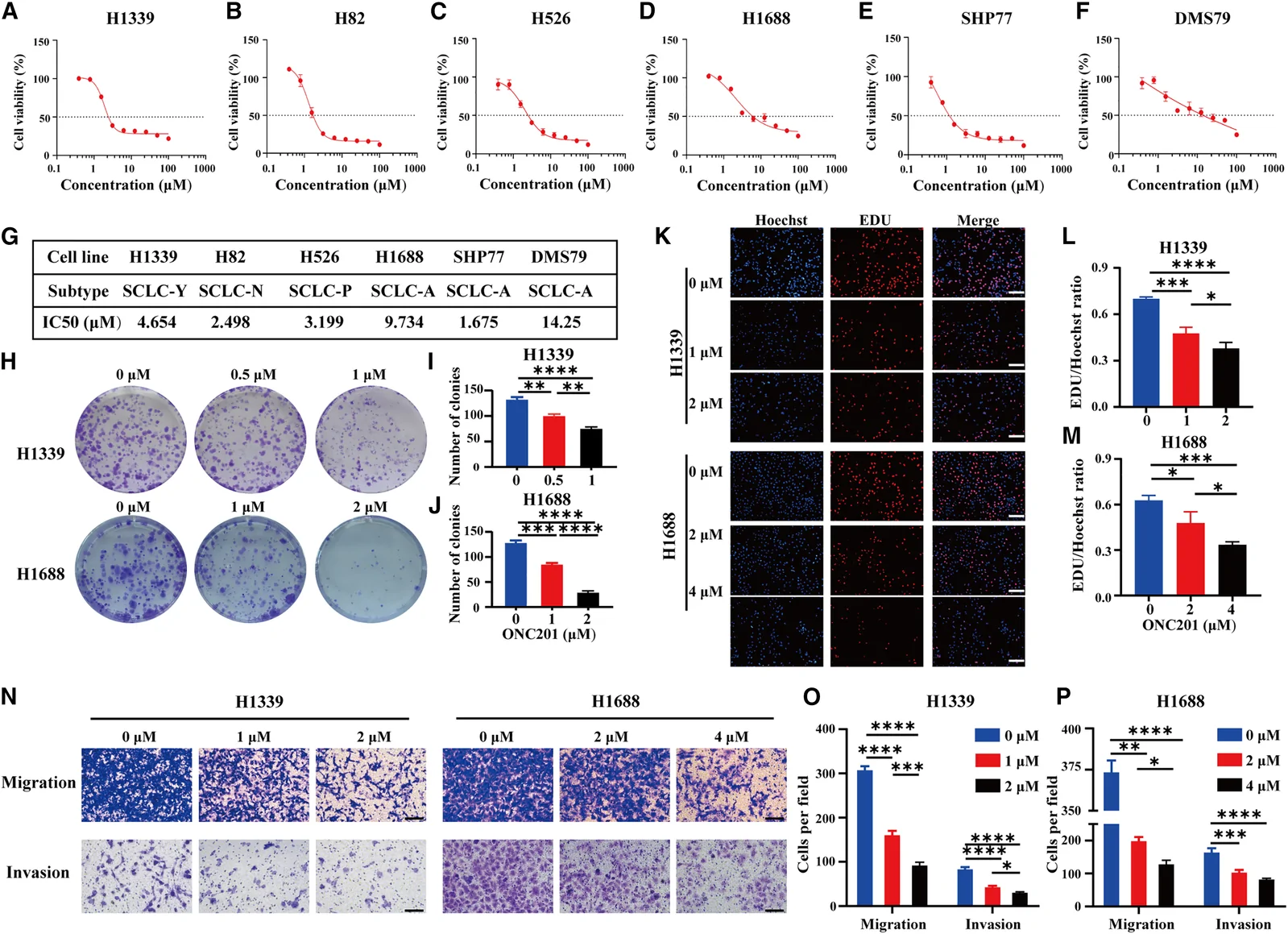
ONC201 suppresses SCLC cell proliferation, migration, and invasion (A–F) Dose-response curves showing the viability of H1339, H82, H526, H1688, SHP77, and DMS79 cells after treatment with 0–100 μM ONC201 for 72 h, as determined by CCK-8 assays. Cell viability was normalized to the DMSO-treated control. (G) Summary of the molecular subtypes and 72 h IC50 values of the indicated SCLC cell lines. (H–J) Representative colony formation images (H) and quantification of colony numbers in H1339 (I) and H1688 (J) cells treated with the indicated concentrations of ONC201. (K–M) Representative EdU staining images (K) and quantification of the EdU/Hoechst ratio in H1339 (L) and H1688 (M) cells after ONC201 treatment for 24 h. Scale bars, 100 μm. (N–P) Representative Transwell migration and invasion images (N) and quantification of migrated or invaded H1339 (O) and H1688 (P) cells after ONC201 treatment for 48 h. Scale bars, 100 μm. Data are shown as mean ± SD from three independent experiments. Statistical significance was determined using one-way ANOVA followed by Tukey’s multiple-comparisons test. ∗*p* < 0.05, ∗∗*p* < 0.01, ∗∗∗*p* < 0.001, and ∗∗∗∗*p* < 0.0001.

Studies have highlighted that SCLC is a highly heterogeneous disease composed of transcription factor-defined subtypes, which contribute to variable treatment sensitivities and the evolution of resistance.^18^^,^^19^ Therefore, to determine whether the antitumor activity of ONC201 extends across molecularly diverse SCLC models, we examined cell viability after 72 h of ONC201 treatment in 6 SCLC cell lines, including H1339, H82, H526, H1688, SHP77, and DMS79. ONC201 reduced cell viability in a dose-dependent manner across these cell lines. Different SCLC cell lines exhibited variable sensitivity to ONC201, with H82 and SHP77 cells showing relatively higher sensitivity, whereas DMS79 cells showed a comparatively lower response (Figures 1A–1G). Consistently, ONC201 reduced cell viability after 48 h of ONC201 treatment, although the inhibitory effect was generally less pronounced than that observed at 72 h, suggesting a time-dependent difference in ONC201 sensitivity (Figure S1). Colony formation and EdU assays conducted using H1339 and H1688 cells showed that sub-IC50 concentrations of ONC201 inhibited cell proliferation in a dose-dependent manner (Figures 1H–1M). Furthermore, Transwell assays showed that ONC201 significantly inhibited the migration and invasion of H1339 and H1688 cells in a dose-dependent manner (Figures 1N–1P). In summary, ONC201 exerts an antitumor effect on SCLC cells *in vitro*, significantly inhibiting their proliferation, migration, and invasion.

### ONC201 promotes cell-cycle arrest and apoptosis in SCLC cells

**Figure 2:**
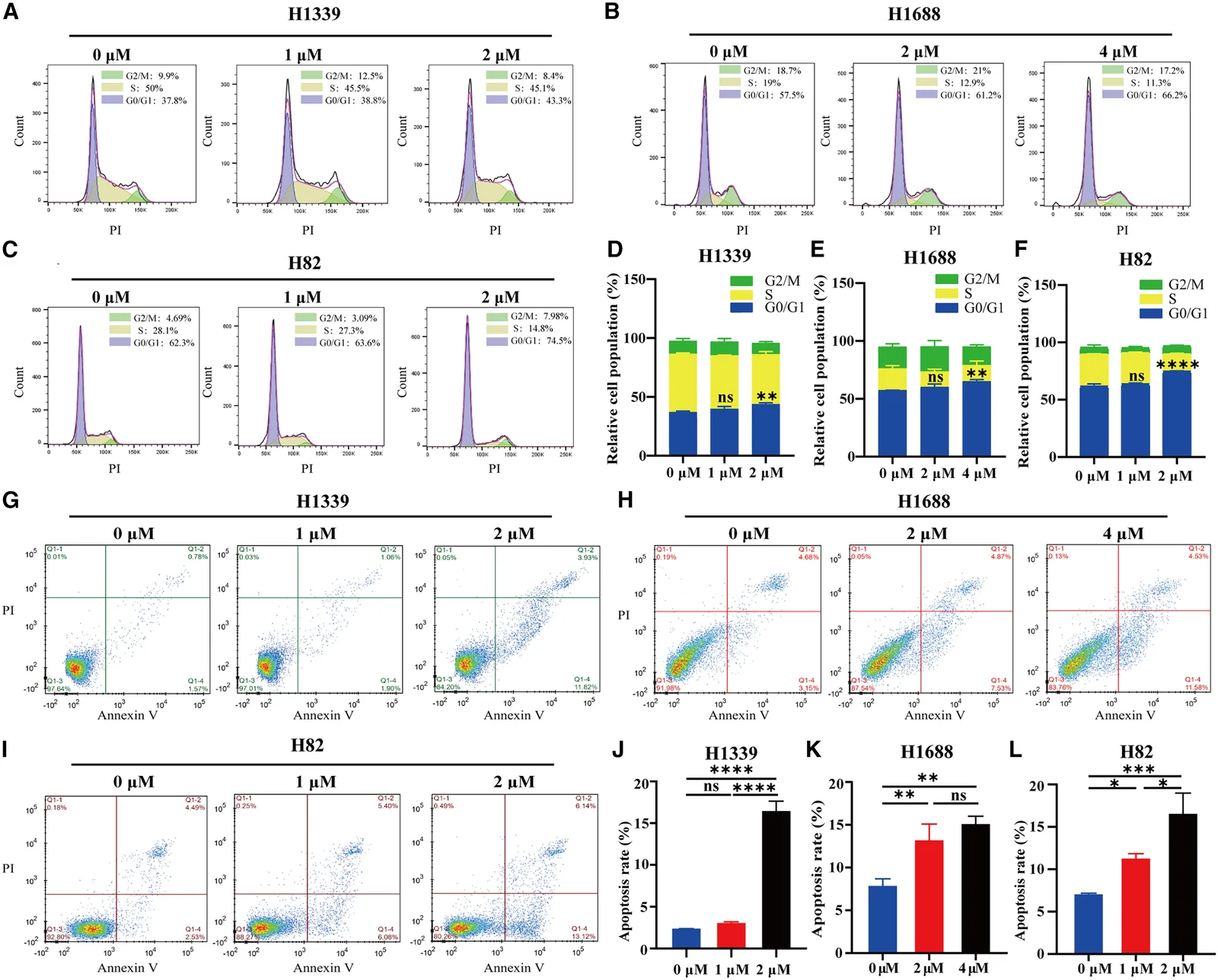
ONC201 induces G0/G1-phase arrest and promotes apoptosis in SCLC cells (A–C) Representative flow cytometry histograms showing cell-cycle distribution in H1339 (A), H1688 (B), and H82 (C) cells after treatment with the indicated concentrations of ONC201 for 48 h, as determined by PI staining. (D–F) Quantification of cell-cycle distribution in H1339 (D), H1688 (E), and H82 (F) cells. (G–I) Flow cytometry plots showing apoptosis in H1339 (G), H1688 (H), and H82 (I) cells after treatment with the indicated concentrations of ONC201 for 48 h, as determined by Annexin V/PI staining. (J–L) Quantification of the overall apoptosis rate in H1339 (J), H1688 (K), and H82 (L) cells. The overall apoptosis rate was calculated as the sum of early and late apoptotic cell populations. Data are shown as mean ± SD from three independent experiments. Statistical significance was determined using one-way ANOVA followed by Tukey’s multiple-comparisons test. ∗*p* < 0.05, ∗∗*p* < 0.01, ∗∗∗*p* < 0.001, and ∗∗∗∗*p* < 0.0001; ns, not significant.

To investigate the impact of ONC201 on cell-cycle progression, we performed flow cytometry with phosphatidylinositol (PI) staining to assess cell-cycle distribution in H1339, H1688, and H82 cells 48 h after ONC201 treatment. The results showed that, compared with the control group, ONC201 treatment significantly increased the proportion of cells in the G0/G1 phase (Figures 2A–2F). Additionally, we performed flow cytometry analysis using Annexin V/PI staining to validate the effect of ONC201 on SCLC cell apoptosis. The results revealed a concentration-dependent increase in the percentage of apoptotic cells in H1339, H1688, and H82 cells (Figures 2G–2L), demonstrating that ONC201 promotes SCLC cell apoptosis in a dose-dependent manner.

### ONC201 enhances the radiosensitivity of SCLC cells

**Figure 3:**
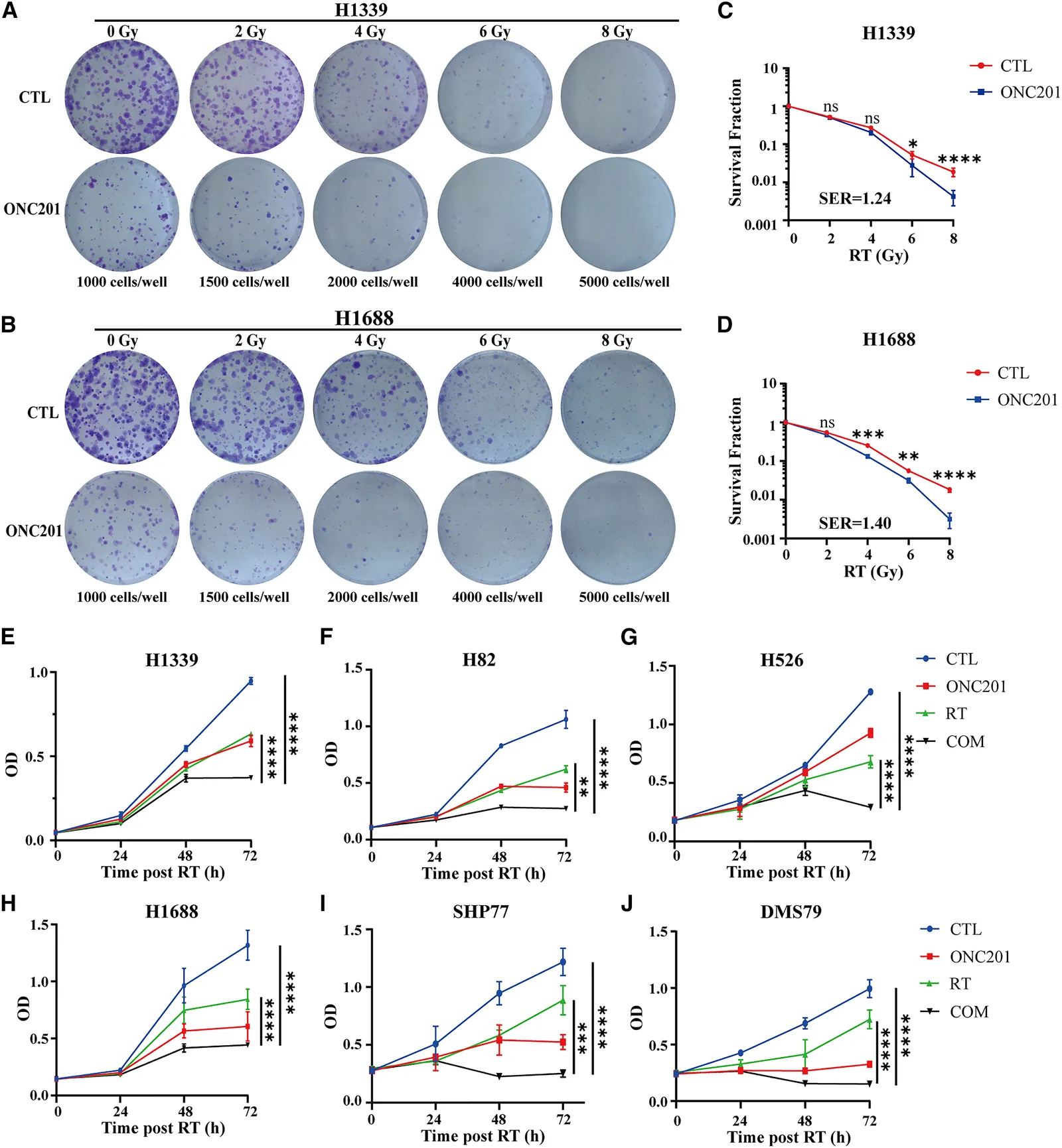
ONC201 enhances the radiosensitivity of SCLC cells (A and B) Representative colony formation images of H1339 (A) and H1688 (B) cells treated with or without ONC201 and exposed to increasing doses of radiation. (C and D) Clonogenic survival curves of H1339 (C) and H1688 (D) cells following irradiation in the presence or absence of ONC201. Survival fractions were calculated from colony formation assays, and the sensitizer enhancement ratio (SER) was determined from the fitted radiation survival curves. (E–J) CCK-8 proliferation assays showing the effects of control treatment, ONC201, radiotherapy (RT), and combined treatment (COM) on H1339 (E), H82 (F), H526 (G), H1688 (H), SHP77 (I), and DMS79 (J) cells at the indicated time points after 4 Gy irradiation. Data are shown as mean ± SD from three independent experiments. Survival fractions at 2–8 Gy were log10-transformed and analyzed using two-way repeated-measures ANOVA followed by Šídák’s multiple-comparisons test. Optical density (OD) values at 72 h were analyzed using one-way ANOVA followed by Tukey’s multiple-comparisons test. ns, not significant; ∗*p* < 0.05, ∗∗*p* < 0.01, ∗∗∗*p* < 0.001, and ∗∗∗∗*p* < 0.0001.

Furthermore, to investigate the radiosensitizing effects of ONC201 in SCLC cells, we performed colony formation assays using H1339 and H1688 cells and observed significantly reduced survival fractions in ONC201-treated groups across all radiation doses compared to the controls (Figures 3A–3D). Quantitative analysis revealed that the sensitizer enhancement ratio (SER) of ONC201 was 1.24 for H1339 cells and 1.40 for H1688 cells (Figures 3C and 3D). To further validate the generalizability of ONC201-mediated radiosensitization, we expanded our analysis to six SCLC cell lines representing distinct molecular subtypes, including H1339, H82, H526, H1688, SHP77, and DMS79. ONC201 showed growth-inhibitory activity across these cell lines and consistently enhanced the anti-proliferative effect of radiotherapy (Figures 3E–3J). These results confirmed that ONC201 exhibited a robust radiosensitizing effect in SCLC cells.

### ONC201 promotes radiotherapy-induced cell-cycle arrest and apoptosis

**Figure 4:**
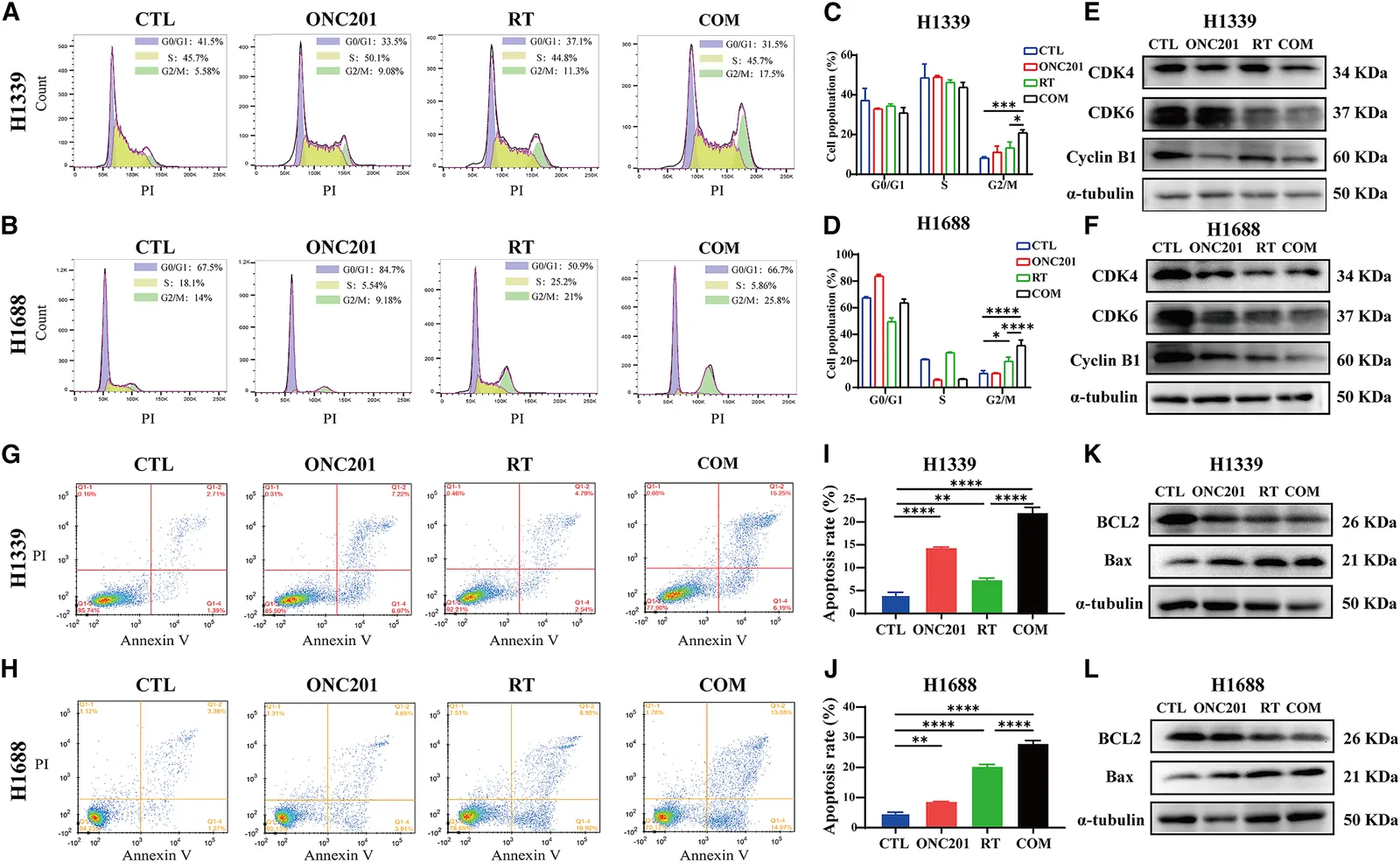
ONC201 enhances radiotherapy-induced cell-cycle arrest and apoptosis in SCLC cells (A and B) Flow cytometry histograms showing cell-cycle distribution in H1339 (A) and H1688 (B) cells after treatment with ONC201 and/or 4 Gy radiotherapy (RT) for 48 h. (C and D) Quantification of cell cycle distribution in H1339 (C) and H1688 (D) cells. (E and F) Western blot images showing the expression of cell-cycle-related proteins CDK4, CDK6, and cyclin B1 in H1339 (E) and H1688 (F) cells after the indicated treatments. (G and H) Representative flow cytometry plots showing apoptosis in H1339 (G) and H1688 (H) cells after treatment with ONC201 and/or 4 Gy RT for 48 h, as determined by Annexin V/PI staining. (I and J) Quantification of the overall apoptotic rate in H1339 (I) and H1688 (J) cells. (K and L) Western blot images showing the expression of apoptosis-related proteins BCL2 and Bax in H1339 (K) and H1688 (L) cells after the indicated treatments. Data are shown as mean ± SD. Cell-cycle distribution was analyzed separately for each phase using one-way ANOVA followed by Tukey’s multiple-comparisons test. Apoptotic rate was analyzed by one-way ANOVA followed by Tukey’s multiple-comparisons test. ∗*p* < 0.05, ∗∗*p* < 0.01, ∗∗∗*p* < 0.001, and ∗∗∗∗*p* < 0.0001.

Previous studies have reported that radiotherapy can activate the DNA damage response and trigger the G2/M checkpoint, resulting in significant cell-cycle arrest at the G2/M phase.^20^^,^^21^ Therefore, we investigated the effects of ONC201 on cell-cycle progression following radiotherapy. Flow cytometry analysis showed that radiotherapy increased the proportion of cells in the G2/M phase compared with the control group, and this effect was further enhanced in the combination group compared with the radiotherapy group (Figures 4A–4D). It was further confirmed by western blot analysis that, compared with radiotherapy alone, the combination group showed markedly decreased expression of key cell-cycle regulators, including cyclin-dependent kinase 4 (CDK4), CDK6, and cyclin B1 (Figures 4E and 4F). CDK4/6 are central drivers of cell-cycle progression, whereas cyclin B1 (in complex with CDK1) is essential for the G2/M transition^22^^,^^23^; therefore, downregulation of these proteins supports enhanced cell-cycle arrest in the combination group. Additionally, we confirmed that the combination of ONC201 and radiotherapy significantly promoted SCLC cell apoptosis (Figures 4G–4J), as evidenced by increased expression of pro-apoptotic effector Bax^24^ and decreased anti-apoptotic effector Bcl-2^25^ expression in the combination group (Figures 4K and 4L). Furthermore, we investigated the role of ONC201 in radiotherapy-induced DNA damage. The expression of γ-H2AX was not significantly higher in the ONC201 group than in the control group 1 h or 24 h post-radiotherapy, whereas the expression of γ-H2AX in the combination group was significantly higher than that in the radiotherapy group 24 h post-radiotherapy (Figure S2). These results suggest that ONC201 may delay DDR or sustain radiotherapy-induced DNA damage after radiotherapy.^15^^,^^26^

### ONC201 enhances SCLC radiosensitivity through suppressing the FAK signaling pathway

**Figure 5:**
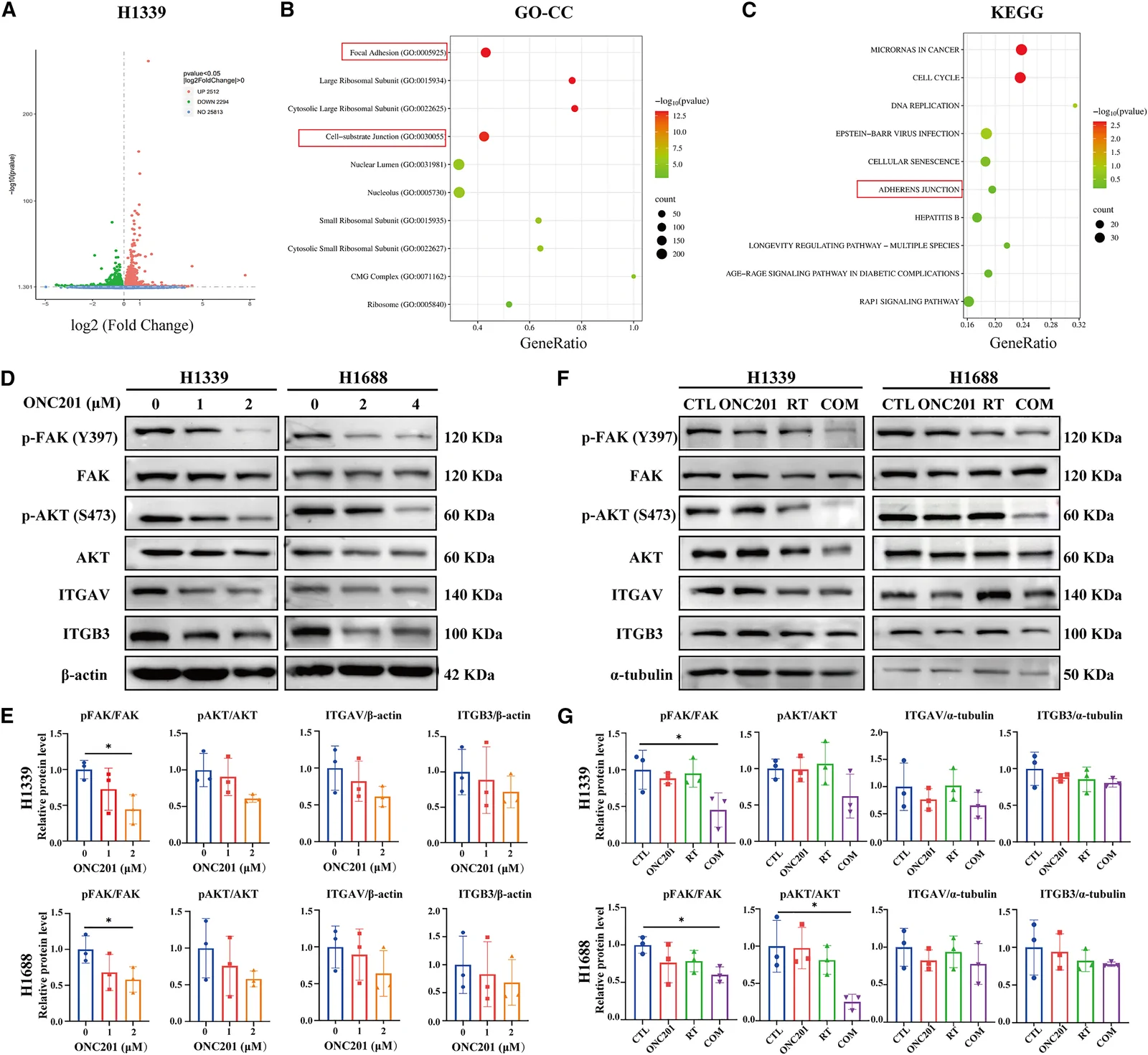
Focal adhesion-related signaling is involved in ONC201-mediated radiosensitization (A) Volcano plot showing differentially expressed genes (DEGs) in the combination treatment (COM) group compared with the radiotherapy (RT) group based on bulk RNA-seq analysis. (B) GO cellular component (GO-CC) enrichment analysis showing the top 10 enriched cellular components in the COM group compared with the RT group. Dot size represents the number of enriched genes, and dot color indicates the enrichment significance, expressed as −log_10_(adjusted *p* value). GeneRatio represents the proportion of enriched genes associated with each term or pathway. (C) KEGG pathway enrichment analysis showing the top 10 downregulated pathways in the COM group compared with the RT group. (D and E) Representative western blot images (D) and corresponding quantification (E) of p-FAK/FAK, p-AKT/AKT, ITGAV/β-actin, and ITGB3/β-actin after ONC201 treatment (48 h). (F and G) Representative western blot images (F) and corresponding quantification (G) of these proteins after 4 Gy RT and/or ONC201 treatment (48 h). Data are shown as mean ± SD from three independent experiments. Statistical significance was determined using one-way ANOVA followed by Tukey’s multiple-comparisons test. ∗ *p* < 0.05. CTL, control; RT, radiotherapy; COM, combination treatment.

To further elucidate the molecular mechanisms underlying ONC201-mediated radiosensitization, we performed bulk RNA-seq analysis of ONC201- and/or radiotherapy-treated H1339 cells. Differentially expressed gene (DEG) analysis revealed that the combination group exhibited 2,512 upregulated and 2,294 downregulated genes compared to the radiotherapy group (Figure 5A). Additionally, principal component analysis (PCA) showed the intra-group reproducibility and the reliability of the experimental results (Figure S3A). Compared with the radiotherapy group, the combination treatment group showed prominent enrichment of adhesion-related terms and pathways. Specifically, focal adhesion ranked as the top enriched Gene Ontology cellular component (GO-CC) term, and cell-substrate junction and adherens junction were also identified among the top enriched GO-CC terms and Kyoto Encyclopedia of Genes and Genomes (KEGG) pathways, respectively. In addition, KEGG analysis revealed enrichment of cell cycle- and DNA replication-associated pathways, in line with our previous results showing that ONC201 promoted cell-cycle arrest following radiotherapy (Figures 5B and 5C). Also of note, we performed gene set enrichment analysis (GSEA), which revealed a significant downregulation of cell adhesion molecules (NES = −1.27, *p* = 0.02) and negative regulation of cell-cycle arrest (NES = −1.67, *p* = 0.04), whereas DNA replication showed a nonsignificant downward trend (NES = −1.32, *p* = 0.15) in the combination group compared with the radiotherapy group (Figures S3B–S3D). These findings indicate that ONC201-mediated radiosensitization may be associated with the regulation of cell adhesion structures and cell-cycle progression.

Focal adhesions are critical structures that mediate cell-matrix adhesion and are primarily composed of integrin receptors, cytoskeletal proteins, and multiple signaling transducers.^27^ As a pivotal effector of extracellular matrix (ECM) signaling, FAK not only regulates cell adhesion, migration, proliferation, and survival by modulating the phosphoinositide 3-kinase-protein kinase B (PI3K-AKT) and mitogen-activated protein kinase (MAPK) pathways but also has been demonstrated to critically mediate tumor resistance to radiotherapy, chemotherapy, and immunotherapy.^28^^,^^29^^,^^30^ Therefore, we hypothesized that ONC201 enhances SCLC radiosensitivity by modulating FAK signaling. Western blot analysis showed that ONC201 treatment reduced p-FAK/FAK levels in H1339 and H1688 cells, while p-AKT/AKT, ITGAV, and ITGB3 showed decreasing trends (Figures 5D and 5E). The combination treatment showed decreased p-FAK/FAK levels compared with the radiotherapy group. In addition, p-AKT/AKT, ITGAV, and ITGB3 also showed decreasing trends (Figures 5F and 5G).

**Figure 6:**
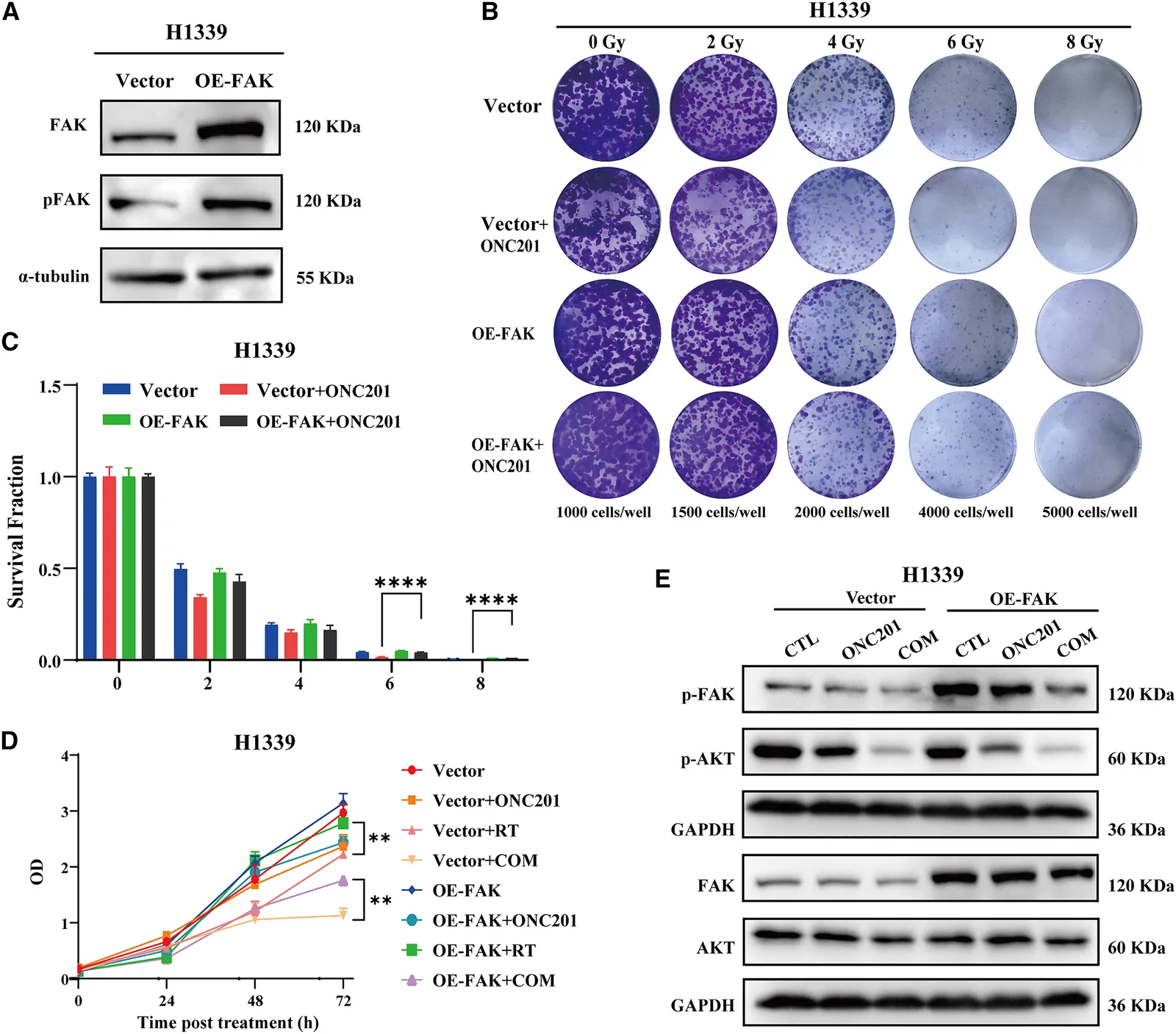
ONC201 enhances SCLC radiosensitivity by suppressing FAK signaling (A) Western blot images confirming FAK overexpression in H1339 cells. α-tubulin was used as the loading control. (B and C) Representative colony formation images (B) and quantification of survival fractions (C) in NC and FAK-overexpressing H1339 cells treated with ONC201 and exposed to increasing doses of radiation. (D) CCK-8 proliferation curves showing the effects of ONC201, radiotherapy (RT), and combination treatment on NC and FAK-overexpressing H1339 cells. (E) Representative western blot images showing the expression of p-FAK, FAK, p-AKT, and AKT in NC and FAK-overexpressing H1339 cells after the indicated treatments. GAPDH was used as the loading control. Data are shown as mean ± SD. Survival fractions at 2–8 Gy were log10-transformed and analyzed using two-way ANOVA followed by Šídák’s multiple-comparisons test, with selected treatment groups compared at each radiation dose. Optical density (OD) values at 72 h were analyzed using two-way ANOVA, with FAK status and treatment as the two factors, followed by Šídák’s multiple-comparisons test for prespecified comparisons. ∗∗*p* < 0.01 and ∗∗∗∗*p* < 0.0001. NC, negative control; OE-FAK, FAK overexpression; RT, radiotherapy; COM, combination treatment.

To further determine whether FAK inhibition is functionally involved in ONC201-mediated radiosensitization, we performed rescue experiments using FAK-overexpressing H1339 cells. FAK overexpression partially restored colony-forming capacity and cell proliferation following ONC201 plus radiotherapy (Figures 6A–6D). Consistently, FAK overexpression partially rescued the ONC201-induced inhibition of the FAK signaling in H1339 cells, as evidenced by the restoration of p-FAK levels after ONC201 or combination treatment (Figure 6E), indicating that enforced FAK expression attenuated the radiosensitizing effect of ONC201. Furthermore, we knocked down FAK in H1339 cells using short hairpin RNA (shRNA) (Figure S4A). Subsequent functional assays demonstrated that while FAK knockdown conferred radiosensitivity, the addition of ONC201 further enhanced radiosensitization (Figures S4B–S4D). As p-FAK levels were not further suppressed in this setting (Figure S4E), this additional effect may be mediated through mechanisms independent of FAK inhibition. These results further support the conclusion that ONC201 enhances radiosensitivity, at least in part, through suppression of FAK signaling.

### ONC201 enhances the antitumor efficacy of radiotherapy *in vivo*

**Figure 7:**
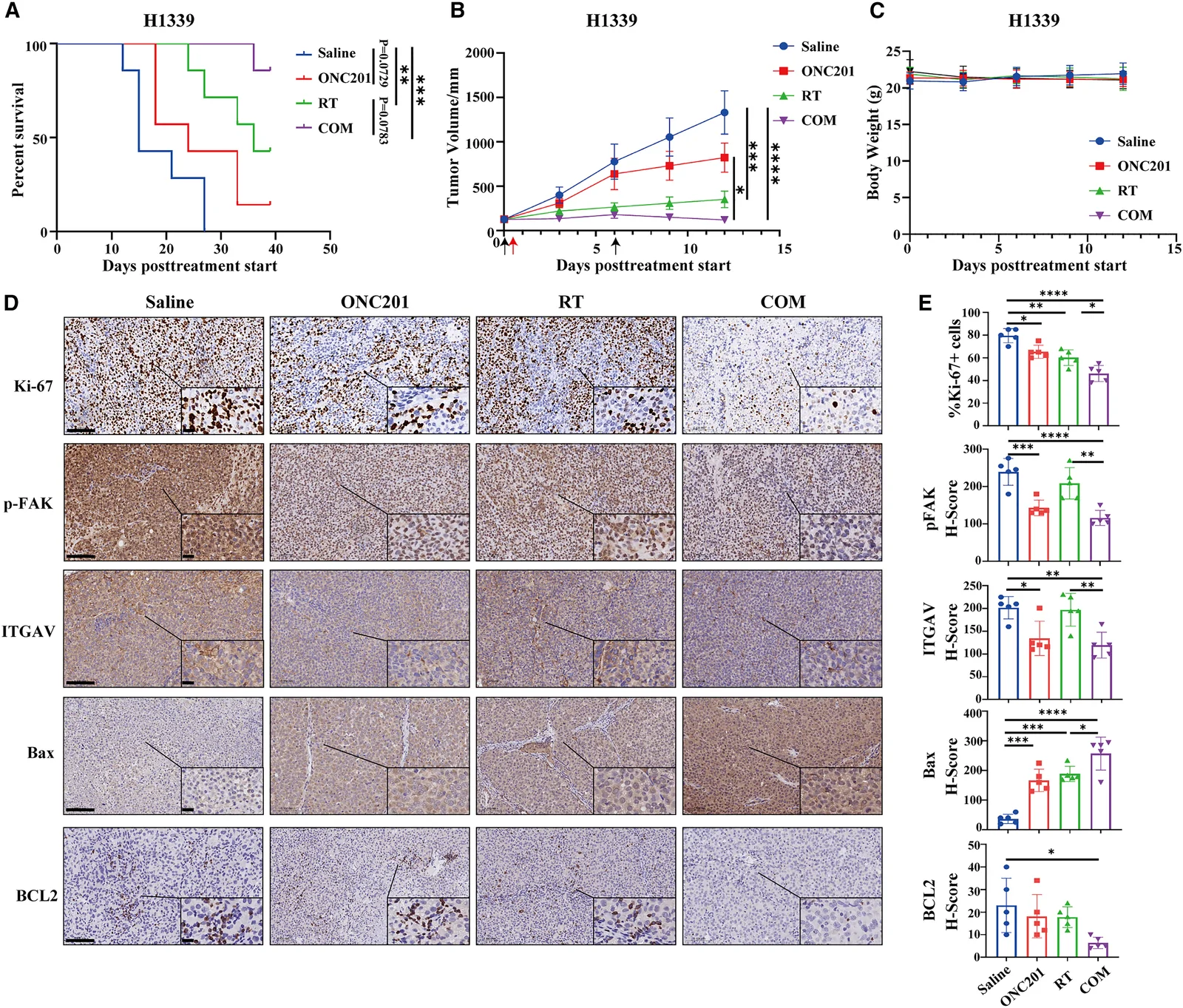
ONC201 enhances the antitumor efficacy of radiotherapy in vivo (A) Kaplan-Meier survival curves of mice bearing H1339 xenografts treated with saline, ONC201, radiotherapy (RT), or combined treatment (COM). ONC201 was administered by oral gavage at 50 mg/kg once weekly, and RT was delivered as a single 5 Gy dose. Red arrows indicate RT, and black arrows indicate ONC201 administration. (B) Tumor volume curves of each group (*n* = 7). Data are shown as mean ± SEM. (C) Body weight of each group. (D and E) Ki-67 was quantified as the percentage of Ki-67-positive cells. The expression levels of p-FAK, ITGAV, Bax, and BCL2 were evaluated using H-scores, calculated as Σ(Pi × i), where Pi represents the percentage of positively stained cells at each staining intensity and i represents the staining intensity score. Scale bars (low-magnification representative fields), 100 μm. Scale bars (high-magnification representative fields), 20 μm. Data are shown as mean ± SD. Survival curves were estimated using the Kaplan-Meier method and compared using the log rank (Mantel-Cox) test. Pairwise comparisons were adjusted for multiple testing using the Holm-Šídák method. Endpoint tumor volumes and IHC quantification data were analyzed using one-way ANOVA followed by Tukey’s multiple-comparisons test. ∗*p* < 0.05, ∗∗*p* < 0.01, ∗∗∗*p* < 0.001, and ∗∗∗∗*p* < 0.0001.

To further confirm the radiosensitizing effect of ONC201 *in vivo*, we established subcutaneous xenograft models using the SCLC cell lines H1339 and H82. In the H1339 xenograft model, we confirmed that the combination of ONC201 and radiotherapy significantly prolonged the survival of mice and inhibited tumor growth (Figures 7A and 7B). We also demonstrated the radiosensitizing effect of ONC201 in the H82 xenograft model, with significantly reduced tumor volume and tumor weight in the combination group (Figures S5A and S5B). In addition, ONC201 and radiotherapy were well tolerated *in vivo*, as the body weights of mice remained stable and no obvious histopathological abnormalities were observed (Figures 7C, S5C, and S5D). Moreover, immunohistochemistry (IHC) analysis of tumor tissues showed that the proportion of Ki-67-positive cells was markedly reduced in the combination group compared with the control and radiotherapy groups (Figures 7D and 7E). Consistently, the combination treatment significantly decreased the expression of p-FAK, ITGAV, and BCL2, while increasing Bax expression (Figures 7D and 7E), further supporting enhanced suppression of tumor proliferation and activation of apoptotic signaling *in vivo*.

## Discussion

SCLC is a highly aggressive neuroendocrine malignancy characterized by rapid progression and poor prognosis.^3^ Curative thoracic chemoradiotherapy remains a treatment backbone for patients with limited-stage SCLC, but remains constrained by rapid relapse.^5^ The current first-line standard for extensive-stage SCLC is platinum-etoposide plus PD-L1 blockade, which improves OS, but most patients still experience early progression.^31^^,^^32^ In the relapsed setting, options such as topotecan and lurbinectedin provide modest response durability, and recent clinical advances (e.g., DLL3-directed T cell engager tarlatamab^33^; atezolizumab plus lurbinectedin as first-line maintenance) underscore the field’s move toward mechanism-based combinations, yet resistance remains common.^34^ Against this background, our findings support ONC201 as a mechanistically distinct candidate for SCLC, particularly in combination with radiotherapy.

ONC201 differs from conventional cytotoxic chemotherapy and immunotherapy by targeting stress and mitochondrial vulnerabilities. Pharmacologically, ONC201 is administered orally and has shown favorable tolerability in early clinical studies, including a first-in-human trial in refractory solid tumors and subsequent studies using a weekly regimen. Importantly, ONC201 is a blood-brain barrier penetrant, a property that could be relevant for SCLC given the high incidence of CNS involvement.^35^ As a first-in-class imipridone compound, ONC201 has been demonstrated to exert broad antitumor activity through the activation of TRAIL/DR5-mediated apoptosis, dopamine receptor D2 antagonism,^36^ mitochondrial ClpP protease activation,^37^ and induction of integrated stress response (ISR).^38^ It has been reported that ONC201 synergized with lurbinectedin to kill SCLC cells by regulating ISR, apoptosis, and DNA breakage-related proteins.^17^ In our study, we demonstrated the antitumor activity of ONC201 in SCLC cells and further verified its regulatory effects on cell-cycle progression and apoptosis. Previous studies have demonstrated that ONC201 acts as a radiosensitizer in prostate cancer by suppressing cell-cycle regulators and DDR molecules.^15^ Our study found that ONC201 monotherapy primarily induced G1-phase arrest in SCLC cells, which is consistent with its role in downregulating CDK4/6 expression and inhibiting the G1/S checkpoint.^38^^,^^39^^,^^40^ In contrast, radiotherapy primarily induced G2/M-phase arrest to allow time for DNA repair. Notably, when ONC201 was combined with radiotherapy, we observed a significant enhancement of the G2/M arrest effect. At the molecular level, the combination therapy not only sustained CDK4/6 suppression but also downregulated cyclin B1, a key driver of the G2/M transition. These results suggest that, through its effects on cell-cycle regulators, ONC201 not only blocked cell-cycle progression from the G1 phase but also profoundly reinforced radiotherapy-induced G2/M arrest by inhibiting cyclin accumulation. This was accompanied by enhanced suppression of proliferation and increased apoptosis, as reflected by elevated pro-apoptotic Bax and decreased anti-apoptotic Bcl-2 expression.

We also confirmed that ONC201 enhances the radiosensitivity of SCLC cells, at least in part, through suppression of FAK signaling. Emerging evidence implicates ECM signaling in the activation of radiotherapy resistance in SCLC.^41^ ECM signaling influences tumor cell responses to radiation through multiple pathways, such as the direct transmission of pro-survival and anti-apoptotic signals via integrin receptors,^42^^,^^43^ serving as a reservoir for secreted mitogens (e.g., VEGF and fibroblast growth factors [FGFs]) to sustain proliferative signaling,^44^^,^^45^ and modulation of tumor reoxygenation dynamics.^46^ As the central orchestrator of ECM signaling, FAK demonstrates aberrant activation that correlates with radioresistance and poor prognosis across multiple malignancies.^29^^,^^30^^,^^47^^,^^48^ Contemporary studies have validated FAK-targeting strategies as effective radiosensitizers in pancreatic, cervical, and NSCLCs.^28^^,^^49^^,^^50^^,^^51^^,^^52^ In the present study, bulk RNA-seq analysis revealed that focal adhesion-related terms and pathways were enriched in the combination group compared with the radiotherapy group, suggesting that adhesion-associated signaling may participate in ONC201-mediated radiosensitization. Further validation showed that ONC201 and the combination treatment reduced FAK activation, as reflected by a decreased p-FAK/FAK ratio. Importantly, FAK overexpression attenuated the radiosensitizing effect of ONC201, whereas FAK knockdown enhanced the response to radiotherapy, supporting a functional contribution of FAK signaling to this process. These findings suggest that ONC201-mediated inhibition of FAK signaling may weaken pro-survival adhesion signaling and thereby increase the susceptibility of SCLC cells to radiotherapy. Nevertheless, because radiotherapy response is regulated by multiple interconnected processes, including DNA damage response, apoptosis, cell-cycle regulation, and tumor microenvironmental factors, FAK inhibition is unlikely to fully account for the radiosensitizing effect of ONC201. Further studies are needed to define how ONC201 modulates DDR and whether FAK signaling interacts with other radiation response pathways in molecularly distinct SCLC subtypes.

In summary, our study demonstrated the therapeutic potential of ONC201 in enhancing radiosensitivity in SCLC via regulating FAK signaling. Our preliminary findings not only provide a new combination strategy for SCLC treatment but also provide mechanistic insight into ONC201-mediated radiosensitization.

### Limitations of the study

This study has some limitations. First, our conclusions are primarily based on *in vitro* experiments and subcutaneous cell line-derived xenograft models; additional validation in patient-derived xenograft models, orthotopic/metastatic tumor models, and organoid models are needed. Second, immune-mediated effects cannot be captured in our current systems; given that immunotherapy is embedded in frontline SCLC management, evaluating ONC201-based combinations in immunocompetent models will be important for assessing their translational potential. Third, although both male and female mice were included, the study was not sufficiently powered to assess sex-specific differences in the antitumor and radiosensitizing effects of ONC201. Lastly, the primary goal of our study was to provide preclinical evidence for the efficacy and mechanism of ONC201 as a novel radiosensitizer in SCLC. As our experiments were conducted in models without acquired resistance, exploring the potential of ONC201 in relapsed or therapy-resistant SCLC remains a critical direction for future investigation.

## Resource availability

### Lead contact

Further information and requests for resources should be directed to and will be fulfilled by the lead contact, Xun Yuan (yuanxun@tjh.tjmu.edu.cn).

### Materials availability

This study did not generate new, unique reagents.

### Data and code availability

• RAW RNA-seq data generated in this study have been deposited in the NCBI Sequence Read Archive (SRA) and are publicly available under BioProject accession PRJNA1321004. The accession number is listed in the key resources table.
• This paper does not report original code.
• Data in this article and any additional information required to reanalyze the data will be provided by the lead contact upon reasonable request.

## Acknowledgments

This work was supported by the 10.13039/100014717National Natural Science Foundation of China (grant no. 82003310, grant no. 82472969) and Noncommunicable Chronic Diseases-National Science and Technology Major Project (2024ZD0519700, 2024ZD0519702).

## Author contributions

Y.Y., data curation, formal analysis, investigation, visualization, writing the original draft and review & editing; Zhaolin Xu, data curation, formal analysis, visualization, review & editing; Ziyang Xu, methodology, validation, review & editing; X.Y. and Q.C., conceptualization, funding acquisition, supervision, review & editing.

## Declaration of interests

The authors declare no competing interests.

## Declaration of generative AI and AI-assisted technologies in the writing process

During the preparation of this work, the authors used ChatGPT and DeepSeek to improve readability and check grammar; the authors reviewed and edited the content as needed and take full responsibility for the content of the publication.

## STAR★Methods

### Key resources table

**Table:** 

REAGENT or RESOURCE	SOURCE	IDENTIFIER
**Antibodies**		

*p*-FAK	Cell Signaling Technology	Cat# 8556; RRID:AB_10891442
*p*-AKT	Cell Signaling Technology	Cat# 4060; RRID: AB_2315049
AKT	Cell Signaling Technology	Cat# 4685; RRID:AB_2225340
Ki-67	Cell Signaling Technology	Cat# 9129; RRID:AB_2687446
CDK4	Cell Signaling Technology	Cat# 12790; RRID:AB_2631166
CDK6	Cell Signaling Technology	Cat# 13331; RRID:AB_2721897
Cyclin B1	Cell Signaling Technology	Cat# 4138; RRID:AB_2072132
BCL2	Cell Signaling Technology	Cat# 15071; RRID: AB_2744528
Bax	Cell Signaling Technology	Cat# 2772; RRID:AB_10695870
γ-H2AX	Cell Signaling Technology	Cat# 9718S; RRID: AB_2118009
FAK	Huabio	Cat# ET1602-25; RRID:AB_3069638
ITGAV	Huabio	Cat# ET1610-15; RRID:AB_3069898
ITGB3	Huabio	Cat# ET1606-49;RRID:AB_3069744
GAPDH	Proteintech	Cat# 60004-1-Ig;RRID:AB_2107436
α-tubulin	Proteintech	Cat# 11224-1-AP; RRID: AB_2210206
β-actin	Proteintech	Cat# 81115-1-RR; RRID:AB_2923704
HRP-conjugated AffiniPure goat anti-rabbit IgG	Proteintech	Cat# SA00001-2; RRID:AB_2722564
Cy3-conjugated Goat anti-Rabbit IgG (H + L)	Abclonal	Cat# AS007; AB_2769089；RRID：AB_2769089

**Bacterial and virus strains**		

ShFAK#1	Genechem	N/A
ShFAK#2	Genechem	N/A
ShFAK#3	Genechem	N/A
OE-FAK	Genechem	N/A

**Chemicals, peptides, and recombinant proteins**		

RIPA	Servicebio	G2002
PMSF	Servicebio	G2008
50×Cocktail	Servicebio	G2006
Phosphatase inhibitors	Servicebio	G2007
TransG A (lentiviral transduction reagent)	GeneChem	N/A
ONC201	Selleck	S7963
Defactinib	MedChemExpress	HY-12289

**Critical commercial assays**		

Enhanced BCA Protein Assay Kit	Beyotime	P0010
Cell-Light EdU Apollo567 *In Vitro* Kit	RioBio	C10310-1
SuperKine™ Maximum Sensitivity Cell Counting Kit-8	Abbkine	BMU106
Annexin V-FITC/PI Apoptosis Detection Kit	Yeasen	40302ES60
Cell Cycle and Apoptosis Analysis Kit	Yeasen	40301ES60
Mycolor One-Step Mycoplasma Detector	Vazyme	D201-01

**Deposited data**		

Raw RNA-sequencing data	This paper	PRJNA1321004

**Experimental models: Cell lines**		

NCI-H1688 cell line	The Cell Bank of the Chinese Academy of Sciences	TCHu154
NCI-H82 cell line	The Cell Bank of the Chinese Academy of Sciences	SCSP-5078
NCI-H526 cell line	The Cell Bank of the Chinese Academy of Sciences	SCSP-5087
SHP77 cell line	ATCC	CRL-2195
DMS79 cell line	ATCC	CRL-2049
NCI-H1339 cell line	Sun Yat-sen University Cancer Center	N/A

**Software and algorithms**		

GraphPad Prism	GraphPad Software	RRID: SCR_002798
FlowJo	BD	RRID: SCR_008520
ImageJ	NIH	RRID:SCR_003070

### Experimental model and study participant details

#### Cell lines

The human small-cell lung cancer cell lines NCI-H1688, NCI-H82, and NCI-H526 were obtained from The Cell Bank of the Chinese Academy of Sciences (Cat# TCHu154, SCSP-5078, and SCSP-5087, respectively), SHP77 and DMS79 were obtained from ATCC (Cat# CRL-2195 and CRL-2049, respectively). NCI-H1339 was kindly provided by Doctor Hui Pan from Sun Yat-sen University Cancer Center. STR authentication reports were provided by the source, and no additional STR profiling was performed in our laboratory after receipt. All cell lines were tested for mycoplasma contamination using a colorimetric isothermal amplification-based mycoplasma detection kit (Cat# D201-01, Vazyme, China) before experimental use and were confirmed to be negative.

#### Cell culture conditions

All cell lines, including NCI-H1339, NCI-H1688, NCI-H82, NCI-H526, DMS79, and SHP77 were cultured in RPMI-1640 medium (Cat# PM150110, Procell, China) with 10% fetal bovine serum (Cat# PR0002515, NEWZERUM) and 1% penicillin-streptomycin solution (Cat# SV30010, Hyclone, China). All the cells were maintained in a humidified incubator at 37°C with a 5% concentration of CO_2_.

#### Animals

BALB/c nude mice (4–6 week old) were obtained from Gempharmatech (Chengdu, China) and were kept in an environment that was pathogen-free. Female mice were used to establish the H1339 xenograft model, whereas male mice were used for the H82 xenograft model. Mice were housed under specific pathogen-free conditions with controlled temperature and humidity and a 12-h light/dark cycle, with free access to food and water. Animals were acclimatized for 7 days before tumor implantation. All animal experiments were approved by the Laboratory Animal Welfare and Ethics Committee of Tongji Hospital, Huazhong University of Science and Technology (TJH-202306043), and were performed in accordance with the relevant institutional guidelines for animal care and use.

### Method details

#### CCK-8

Cell viability was assessed using the CCK-8 assay. Cells were seeded in 96-well plates with (3–5) ×10^3^ cells/well. Following adherence, medium containing a 0–100 μM concentration of ONC201 was added. After 48 or 72 h, CCK-8 was added, and optical density (OD) at 450 nm was measured using a microplate reader. IC50 values were calculated from fitted dose–response curves using nonlinear regression after normalization to the DMSO-treated control. CCK-8 was also used for the proliferation assay, and OD was measured at 24, 48, and 72 h post-radiotherapy.

#### 5-Ethynyl-2′-deoxyuridine (EdU)

Cells were seeded at the same density as described above for the cell viability assay. After treatment with different concentrations of ONC201 for 24 h, 50 μM EdU was added and incubated at 37 °C for 2 h. Following fixation (4% paraformaldehyde), neutralization (2 mg/mL glycine), and permeabilization (0.5% Triton X-100), the cells were incubated in Apollo staining solution at room temperature for 30 min. Finally, Hoechst 33342 was used for nuclear staining, and the cells were observed under a fluorescence microscope.

#### Transwell assay

Cell migration and invasion were assessed using Transwell chambers (8 μm pore size, Corning, USA). Cells with 200 μL FBS-free medium were placed in the upper chamber, which was (invasion) or was not (migration) pretreated with 40 μL Matrigel (Corning Incorporated, USA) for 30–40 min. The lower chamber was filled with 700 μL cell culture medium containing 10% FBS. After incubation for 48 h, the chambers were fixed and stained with 0.5% crystal violet. The cells that passed through the membrane were observed under an inverted light microscope.

#### Cell cycle and apoptosis assay

For the cell cycle assay, cells were collected 48 h after ONC201 or RT treatment and fixed with 70% ethanol at 4°C overnight. The cells were then washed twice with PBS and incubated in the dark at room temperature for 30 min in a staining solution containing PI and RNase. Subsequently, the samples were washed and analyzed using a flow cytometer provided by the Experimental Medicine Center, Tongji Hospital, Tongji Medical College, Huazhong University of Science and Technology. FlowJo _v10.8.1 was used for data analysis.

For cell apoptosis analysis, cells were collected and incubated in binding buffer containing Annexin V and PI dye at room temperature for 15 min. Single-stain controls and a blank control were also included. Then the samples were washed and placed on ice for flow cytometry.

#### Western blotting

After the different treatments, the samples were washed and lysed with RIPA buffer containing 1% PMSF, 1% phosphatase inhibitors and 50X cocktail (Servicebio, China) on ice for 30 min. The mixture was sonicated, centrifuged, diluted with loading buffer, and the protein concentration was determined using a BCA protein assay kit (Beyotime, China). Proteins were separated by 10% or 12% sodium dodecyl sulfate-polyacrylamide gel electrophoresis (SDS-PAGE, Yeasen, China) and electrophoretically transferred to polyvinylidene difluoride (PVDF) membranes (Millipore, USA). After blocking with 5% BSA for 1 h, the membranes were incubated overnight at 4°C with the primary antibody. The primary antibodies were listed as: AKT, *p*-AKT, GAPDH, α-tubulin, β-actin, *p*-FAK, FAK, ITGAV, ITGB3, CDK4, CDK6, Cyclin B1, Bax, BCL2. Thereafter, secondary antibody (HRP-conjugated AffiniPure goat anti-rabbit IgG), were applied for 1 h at room temperature, and the protein blots were imaged using the Syngene G: BOX Chemi XT4 imaging system (UK). Band intensities were quantified using ImageJ software. The relative expression levels of target proteins were normalized to the corresponding loading control. For phosphorylated proteins, the relative phosphorylation levels were calculated by normalizing the intensity of phosphorylated proteins to their corresponding total protein levels.

#### RNA-sequencing

H1339 cells were collected at 48 h after ONC201 and/or radiotherapy, and five samples were prepared for each group. After isolation, RNA samples were purified for library preparation and sequenced on the Illumina HiSeq platform (Novogene, Beijing, China). Raw sequencing data were processed using the NovoMagic platform, including quality control, gene expression quantification, PCA, and differential expression analysis. The DEG lists were then uploaded to an online bioinformatics platform (Enrichr, https://maayanlab.cloud/Enrichr/) for GO and KEGG enrichment analyses. GO cellular component (GO-CC) and KEGG pathway enrichment results were visualized as bubble plots using online bioinformatics platform (https://www.bioinformatics.com.cn/).

#### Colony formation assay

The cells were distributed in 6-well plates at a density of 1000 cells/well (0 Gy), 1500 cells/well (2 Gy), 2000 cells/well (4 Gy), 4000 cells/well (6 Gy), and 5000 cells/well (8 Gy). After adherence, the cells were pretreated with ONC201 for 1 h before RT, and ONC201 was washed 48 h after RT. Subsequently, the culture was continued for 10-14 days to observe cell colonies. Finally, the cells were fixed with 4% paraformaldehyde for 15 min and stained with crystal violet. Colonies (>50 μm in diameter and containing ≥50 cells) were counted after 10–14 days. The survival fraction (SF) was calculated using the following formula: SF = Survival fraction= (Number of colonies in the treated group/Number of cells plated in the treated group)/(Number of colonies in the control group/Number of cells plated in the control group). The survival curves were fitted using the single-hit multi-target model: SF = 1 − (1 − e^∧^−D/D0)^∧^N. The SER was calculated as D0(control)/D0(ONC201).

#### Immunofluorescence (IF)

The cells were inoculated into 24-well plates and treated with ONC201 and/or radiotherapy. One hour or 24 h after 4 Gy irradiation, the cells were fixed (4% paraformaldehyde), permeabilized (0.5% Triton X-100), blocked (5% BSA), and incubated with a γ-H2AX primary antibody (1:1000) at 4°C overnight. Subsequently, the cells were incubated with Cy3-conjugated Goat anti-Rabbit IgG (1:300) for 1 h. Finally, after 5 min of DAPI staining, the samples were observed under a fluorescence microscope.

#### Lentiviral transfection

FAK (PTK2) knockdown was achieved using lentiviral short hairpin RNA (shRNA). Lentiviral particles targeting FAK (sh-FAK; shFAK#1, shFAK#2, and shFAK#3) and a non-targeting control (sh-NC), along with the manufacturer’s transduction reagent (TransG A), were purchased from GeneChem (Shanghai, China). A FAK-overexpression lentiviral vector or corresponding control vector were also purchased from GeneChem (Shanghai, China). Viral titers provided by the manufacturer were 1.8×10^9^ transducing units (TU)/mL (shFAK#1), 1.1×10^9^ TU/mL (shFAK#2), 1.5×10^9^ TU/mL (shFAK#3), and 2.9×10^9^ TU/mL (OE-FAK). Cells were seeded in 6-well plates at 2.5×10^5^ cells/well and infected at MOI = 10, according to the manufacturer’s protocol. Briefly, transduction was performed in complete medium supplemented with TransG A (40 μL/well). After 16 h, the transduction medium was replaced with a fresh complete medium. Puromycin (2 μg/mL) was added 72 h post-infection to select for stable knockdown cell lines. Knockdown efficiency was confirmed by western blotting before subsequent experiments.

#### Xenograft models and treatment

1 × 10^7^ H1339 cells or H82 cells suspended in 100 μL PBS were subcutaneously injected into the right axillary region of the mice and tumor volume was monitored (V = L × W^2^/2, where L is length and W is width). When the tumor volume reached 100 mm3, the mice were randomly divided into four groups. Saline and 50 mg/kg ONC201 were administered orally (once a week). Radiotherapy was performed using an RS2000 Plus X-ray biological irradiator (Rad Source Technologies, USA). Mice were immobilized and irradiated with a single dose of 5 Gy. Body weight and tumor volume were measured every three days. Mice were euthanized when the tumor volume reached 2000 mm^3^. Euthanasia was performed by cervical dislocation under deep anesthesia. Tumors and major organs were collected, fixed, and embedded in paraffin wax for subsequent histological analysis.

#### Immunohistochemistry (IHC)

Following standard H&E staining procedures of baking, deparaffinization, and rehydration, tissue sections underwent antigen retrieval in citrate or EDTA-Tris buffer (95°C–98°C, 15–20 min). Endogenous peroxidase was blocked with 3% H_2_O_2_, and nonspecific binding was reduced with goat serum. Sections were incubated with primary antibody at 4°C overnight, followed by a secondary antibody (1:200) at 37°C for 30 min. DAB was applied for color development, and nuclei were counterstained with hematoxylin. Finally, sections were dehydrated, cleared, and mounted for microscopic observation or whole-slide scanning. Ki-67 was quantified as the percentage of Ki-67-positive cells, and other markers were quantified using H score. H score = Σ (Pi × i), Pi: the percentage of positive cells, i: Staining intensity score (0, 1, 2, 3).

#### Hematoxylin and eosin (H&E)

Following baking at 60°C for 60 min and cooling to room temperature, tissue sections were deparaffinized using xylene substitutes and rehydrated through a graded ethanol series (100%–50%). Nuclei were stained with hematoxylin for 2 min, differentiated briefly in 1% acid alcohol, and rinsed. Cytoplasm was counterstained with eosin for 5 min. Sections were then dehydrated through graded ethanol, cleared in xylene substitute, air-dried, and mounted with neutral resin for microscopic examination or whole-slide scanning.

### Quantification and statistical analyses

Statistical analyses were performed using GraphPad Prism 8 (GraphPad Software, Boston, MA, USA). Data are presented as mean ± SD unless otherwise indicated. The numbers of independent experiments and animals are specified in the corresponding figure legends. Comparisons between two independent groups were performed using an unpaired two-tailed Student’s *t* test. Comparisons among three or more groups at a single time point or experimental endpoint were performed using one-way ANOVA followed by Tukey’s multiple-comparisons test. Experiments involving two independent variables were analyzed using two-way ANOVA followed by Šídák’s multiple-comparisons test for prespecified comparisons. For clonogenic survival assays, survival fractions at 2–8 Gy were log10-transformed before statistical analysis, and treatment groups were compared at each radiation dose using two-way ANOVA followed by Šídák’s multiple-comparisons test. Kaplan–Meier survival curves were compared using the log rank (Mantel–Cox) test, with [Holm–Šídák/Bonferroni] correction applied to multiple pairwise comparisons. All statistical tests were two-sided, and *p* < 0.05 was considered statistically significant. Statistical significance is indicated as follows: ∗*p* < 0.05, ∗∗*p* < 0.01, ∗∗∗*p* < 0.001, and ∗∗∗∗*p* < 0.0001; ns, not significant.
