# Supplementary material for: ONC201 enhances the radiosensitivity of small-cell lung cancer via inhibition of the FAK signaling pathway
Source: iScience. 2026 Aug 14;29(9):117202. doi: 10.1016/j.isci.2026.117202 (PMC13503089; doi:10.1016/j.isci.2026.117202)
Supplement: Supplementary file 1 [file mmc1.pdf]

iScience, Volume *29*

Supplemental information

ONC201 enhances the radiosensitivity of small-cell lung cancer via inhibition of the FAK signaling pathway

Yuheng Yan, Zhaolin Xu, Ziyang Xu, Xun Yuan, and Qian Chu

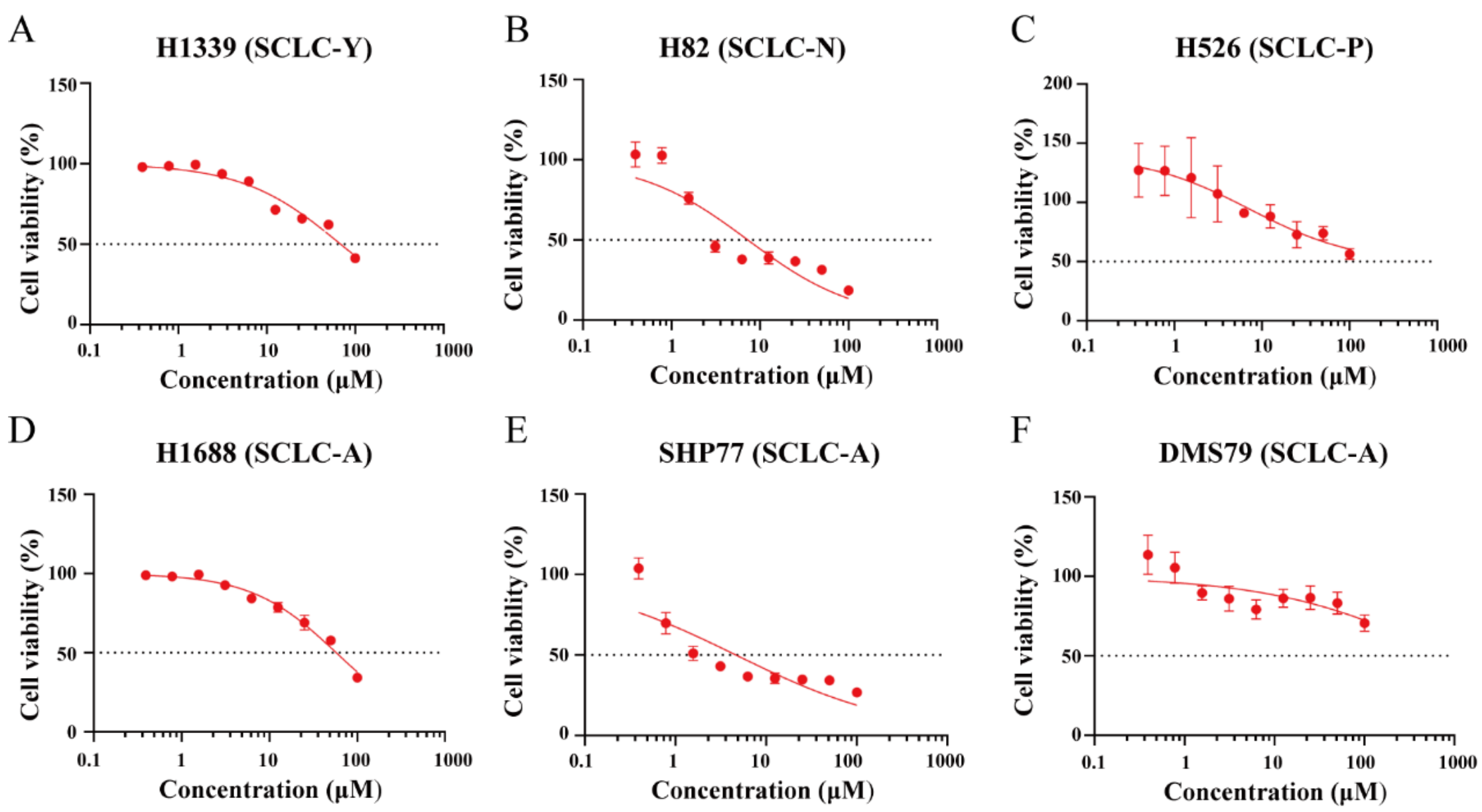

Figure S1. ONC201 inhibits the viability of SCLC cells after 48 h of treatment.

(A–F) Dose–response curves showing the viability of H1339 (A), H82 (B), H526 (C), H1688 (D), SHP77 (E), and DMS79 (F) cells after treatment with the 0-100 μM ONC201 for 48 h, as determined by CCK-8 assays. Cell viability was normalized to the DMSO-treated control. The dotted line indicates 50% cell viability.

Data are shown as mean ± SD from three independent experiments.

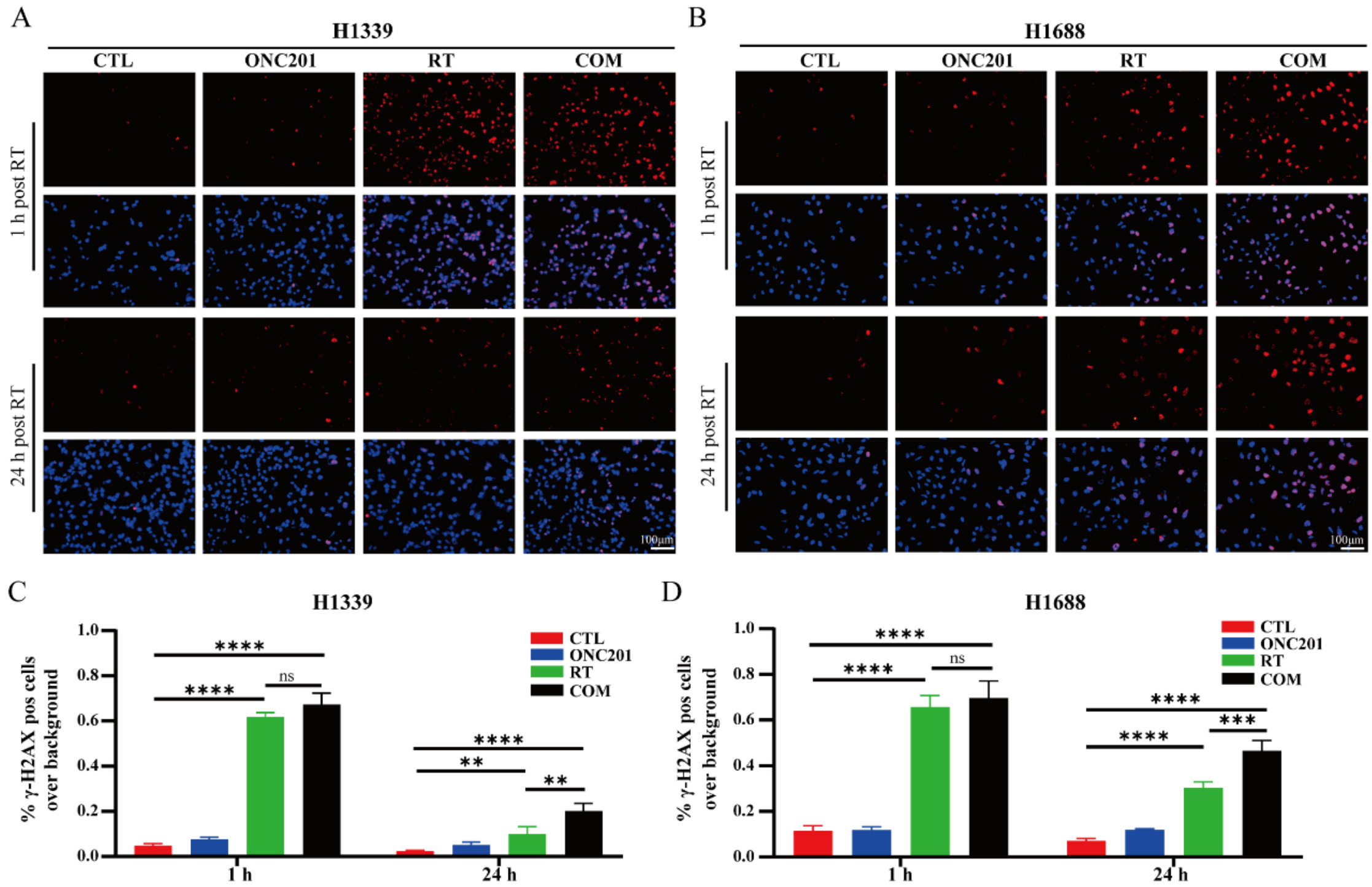

Figure S2. ONC201 inhibits DNA damage repair in SCLC.

(A-B) Immunofluorescence was performed to assess the expression of nuclear γ-H2AX at 1 h and 24 h post-radiotherapy. Nuclei were stained with DAPI (blue), and γ-H2AX was labeled in red. Scale bars, 100 μm.

(C-D) The DNA damage rate was calculated as the ratio of γ-H2AX to DAPI.

Data are shown as mean ±SD. Statistical significance was determined using two-way ANOVA followed by Šídák's multiple-comparisons test for prespecified comparisons among treatment groups at each time point. ** : $P < 0.01$, *** : $P < 0.001$, and **** : $P < 0.0001$; ns: not significant.

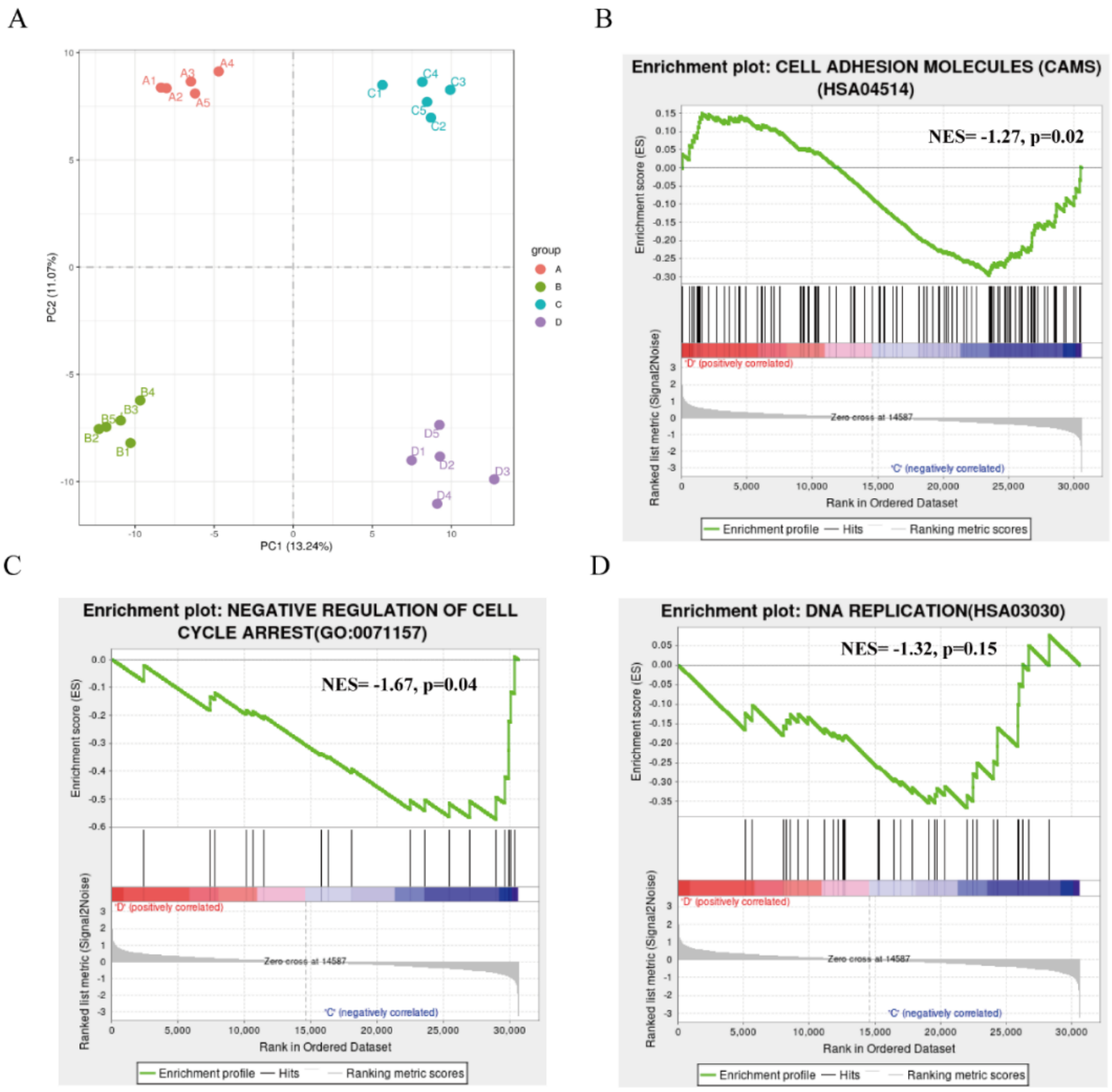

Figure S3. Additional analysis of the RNA-sequencing data.

(A) Principal component analysis (PCA) revealed the intra-group reproducibility and the reliability of the experimental results.

(B-D) Gene Set Enrichment Analysis (GSEA) revealed a significant downregulation of cell adhesion molecules (NES = -1.27, p=0.02), negative regulation of cell cycle arrest (NES=-1.67, p=0.04), and DNA replication (NES = -1.32, p=0.15) in the combination group compared to the radiotherapy group.

A: control group; B: ONC201 group; C: radiotherapy group; D: combination group.

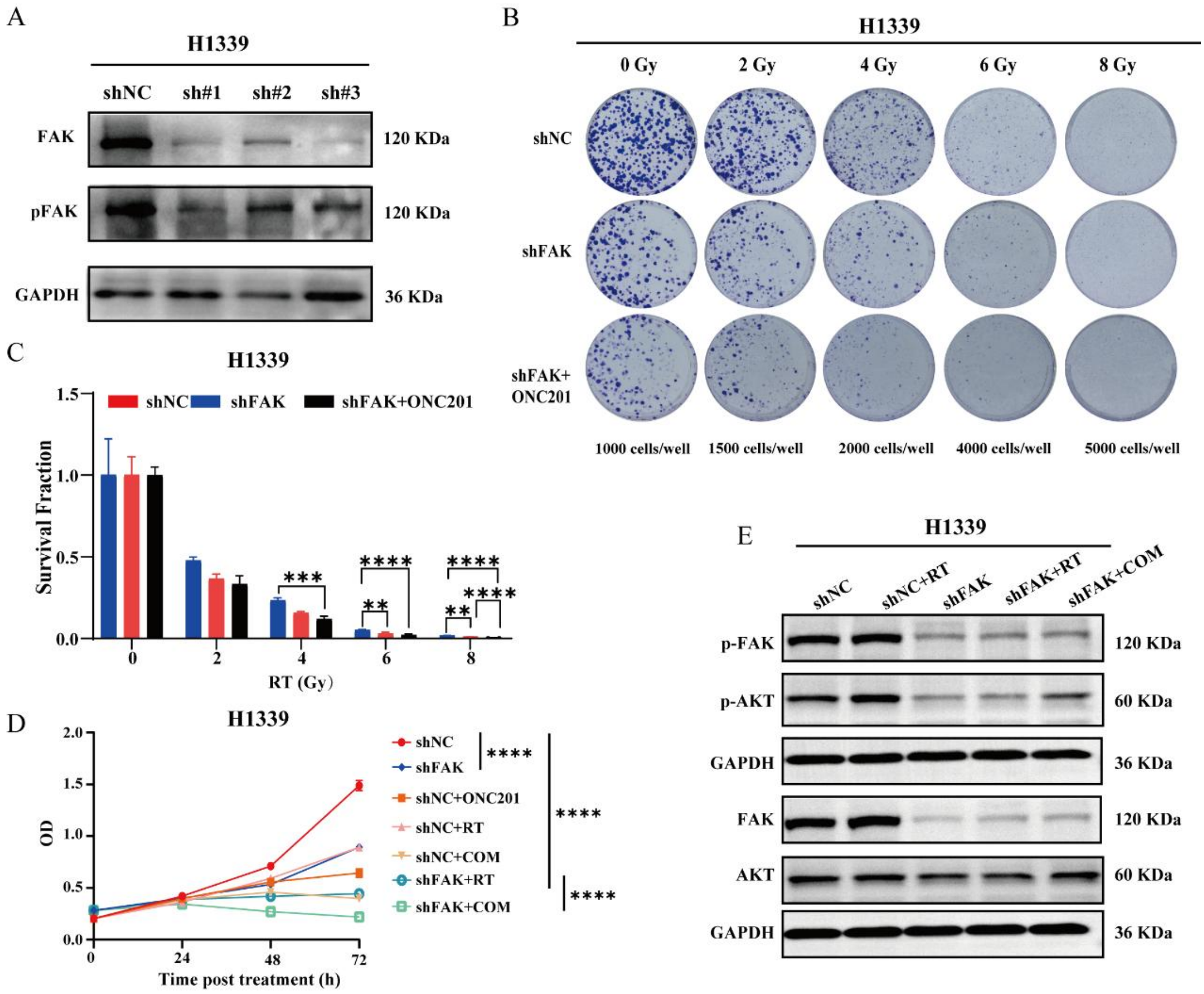

Figure S4. FAK knockdown enhances radiosensitivity in H1339 cells.

(A) Representative western blot images showing the knockdown efficiency of FAK in H1339 cells transduced with shNC or different FAK-targeting shRNAs. GAPDH was used as the loading control.

(B-C) Colony formation images (B) and quantification of survival fractions (C) in shNC and FAK-knockdown H1339 cells treated with or without ONC201 and exposed to increasing doses of radiation.

(D) CCK-8 proliferation curves showing the effects of FAK knockdown, ONC201, radiotherapy (RT), and combined treatment on H1339 cell proliferation at the indicated time points.

(E) Representative western blot images showing the expression of p-FAK, FAK, p-AKT, and AKT in H1339 cells after the indicated treatments. GAPDH was used as the loading control. Data are shown as mean ±SD. Survival fractions at 2–8 Gy were log10-transformed and analyzed using two-way ANOVA followed by Šídák's multiple-comparisons test for prespecified comparisons among treatment groups at each radiation dose. OD values at 72 h were analyzed using one-way ANOVA. **：$P < 0.01$， ****：$P < 0.0001$. shNC, short hairpin negative control; shFAK, FAK knockdown; RT, radiotherapy; COM, combination treatment.

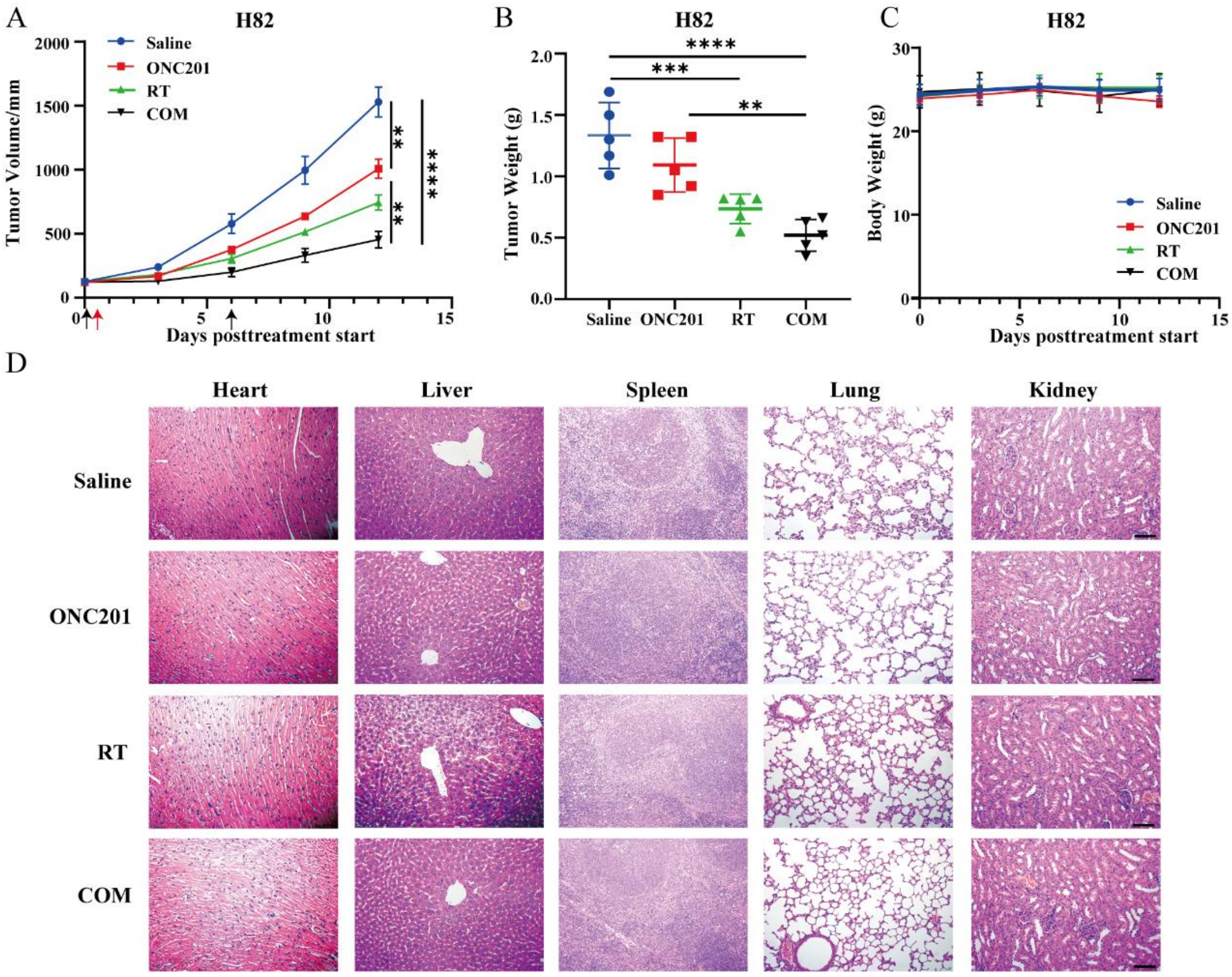

Figure S5. ONC201 synergizes with radiotherapy to suppress the growth of H82 xenograft model.

(A) Tumor volume (Data are shown as mean ± SEM), (B) Tumor weight, and (C) body weight of each group (N=5).

(D) H&E staining of major organs (heart, liver, spleen, lungs, kidneys).

Data in B and C are shown as mean ±SD. Endpoint tumor volumes and tumor weight were analyzed using one-way ANOVA followed by Tukey's multiple-comparisons test. *：*P*＜0.05，**：*P*＜0.01，*** : *P*＜0.001. Scale bars,100 μm.

Data S1: Original western blot images

Figure 4E

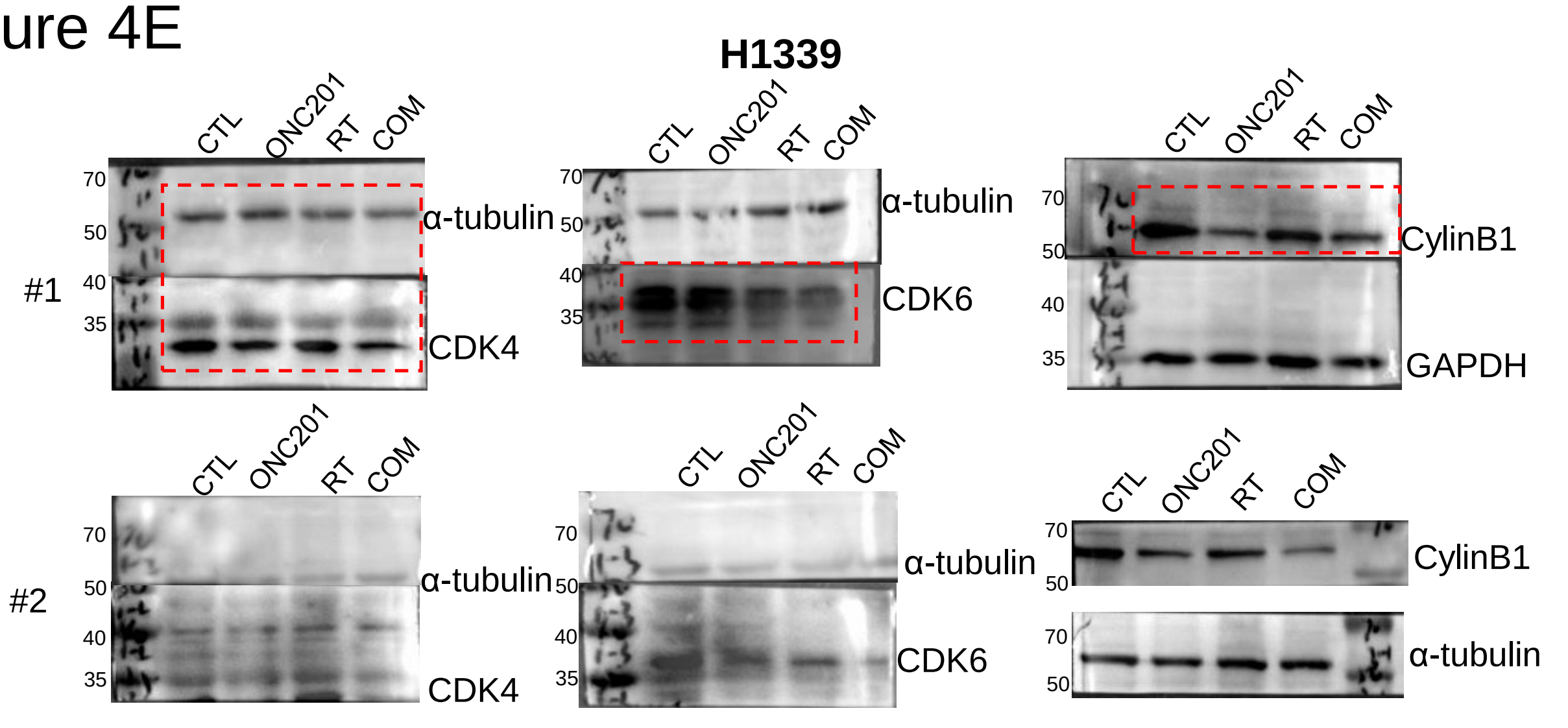

Figure 4F

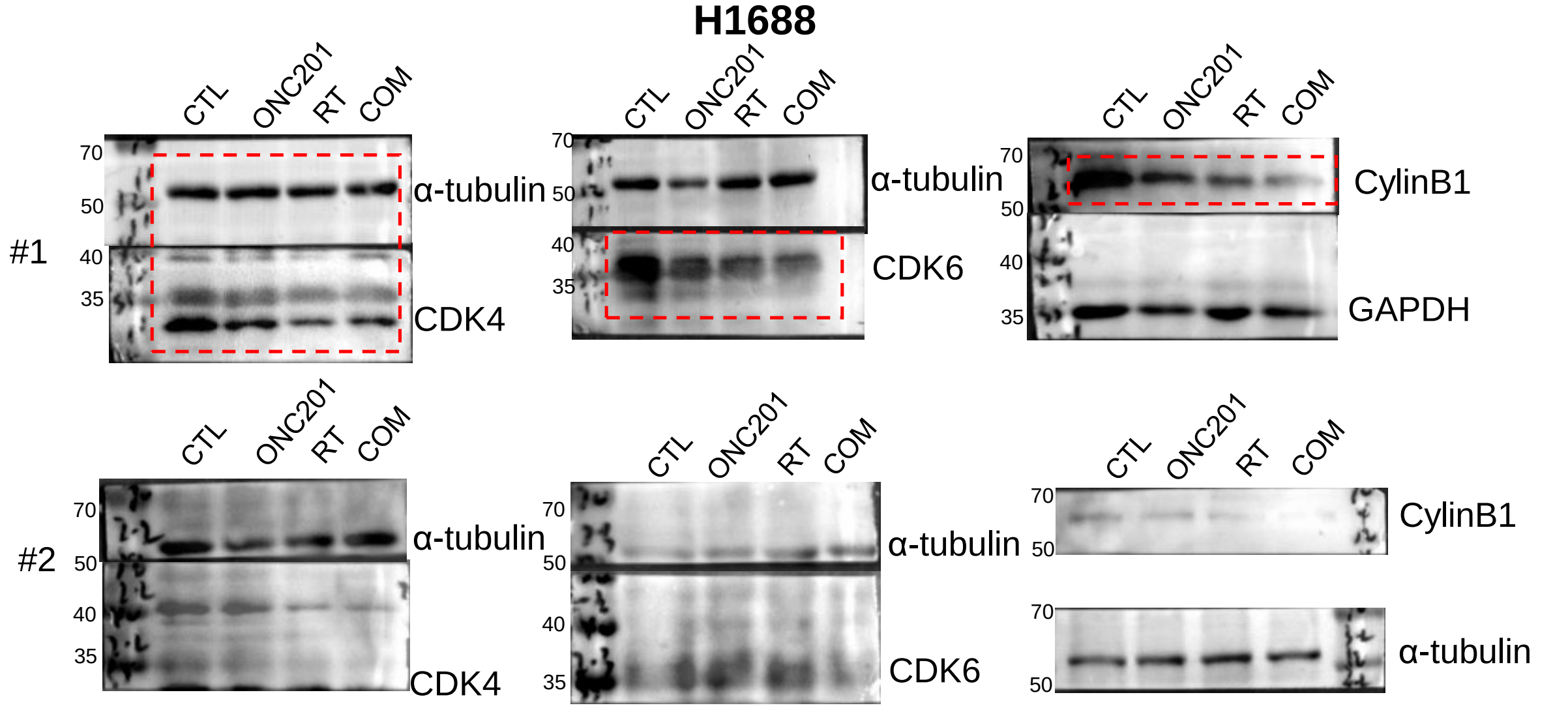

Figure 4K

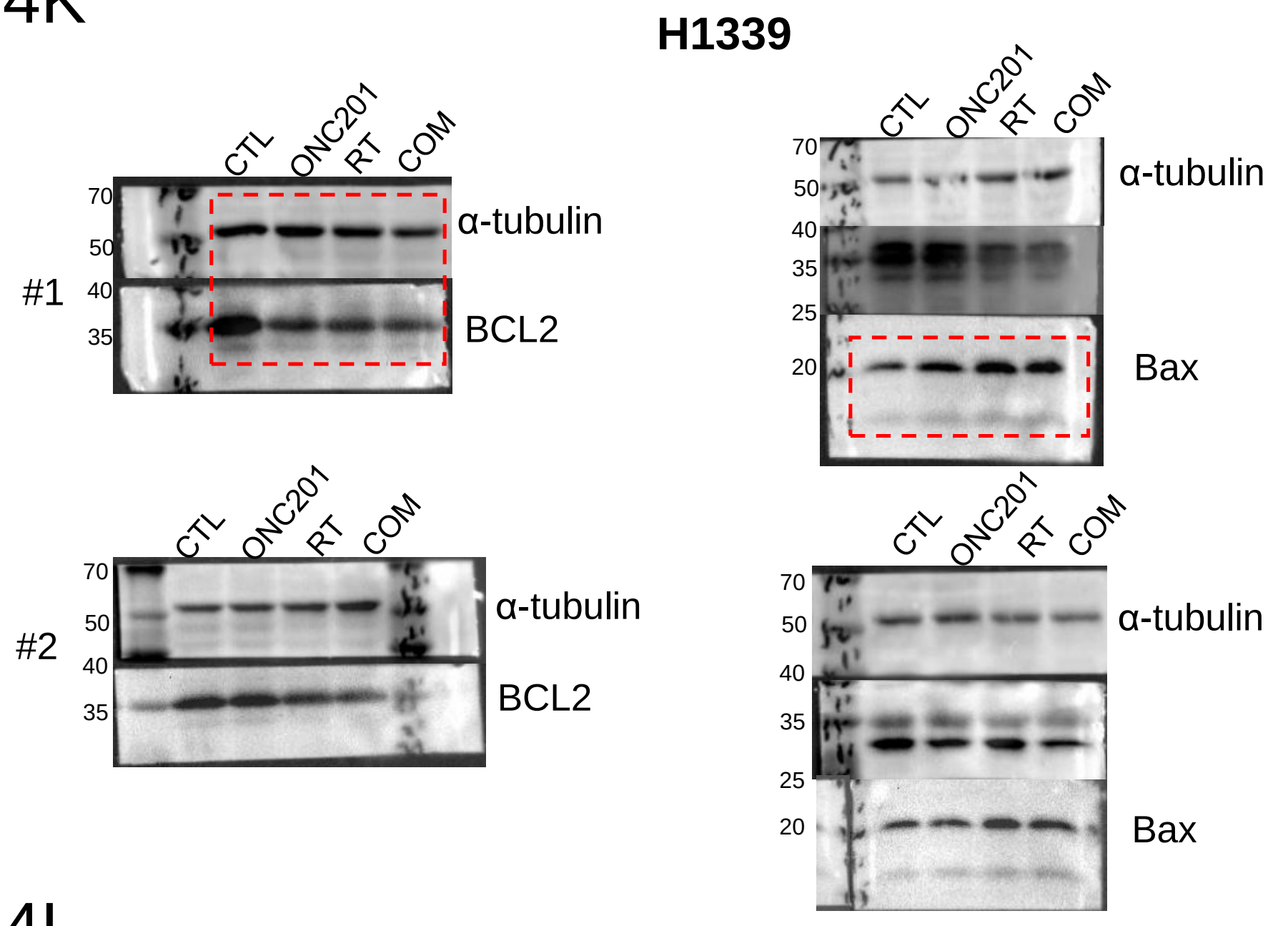

Figure 4L

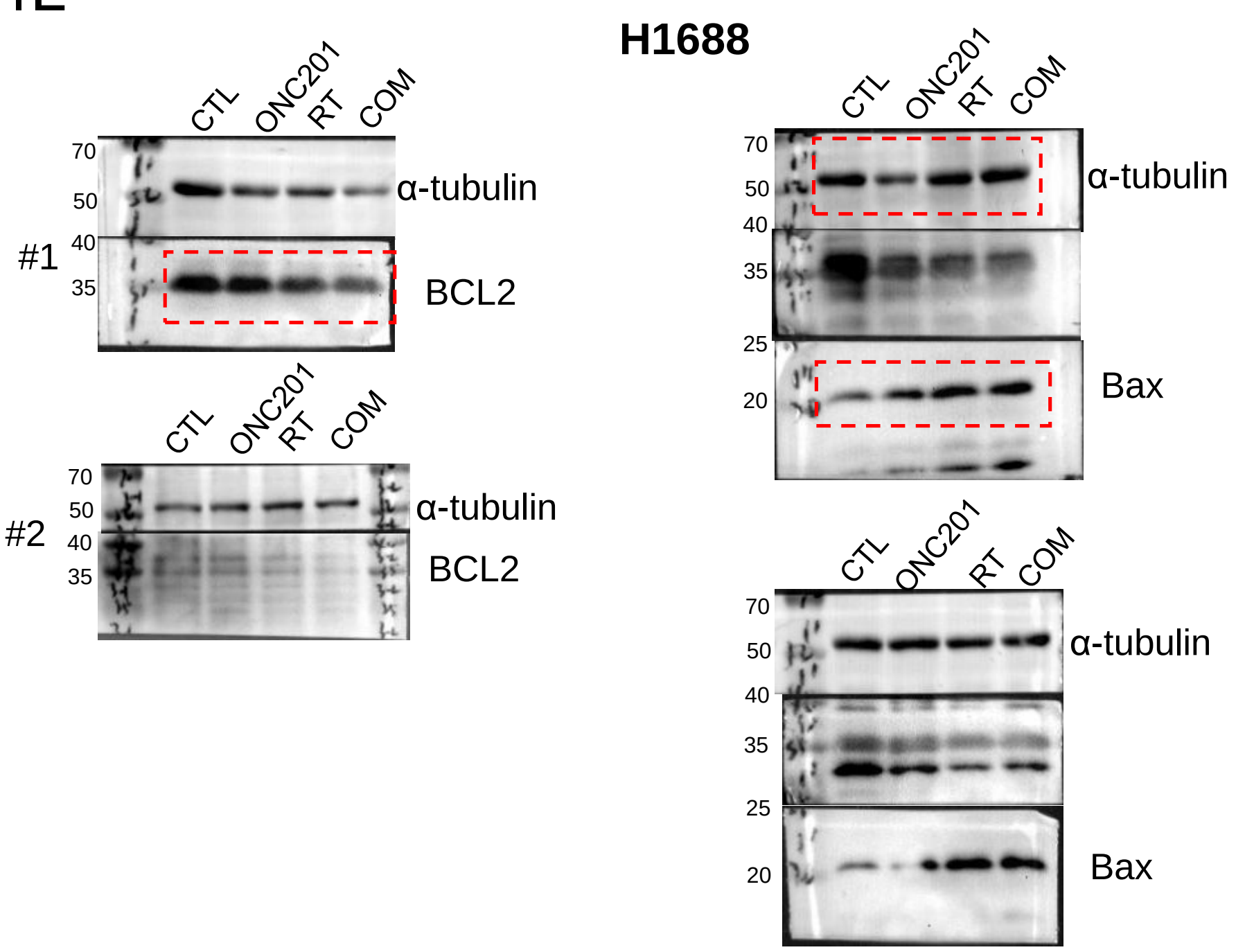

Figure 5D

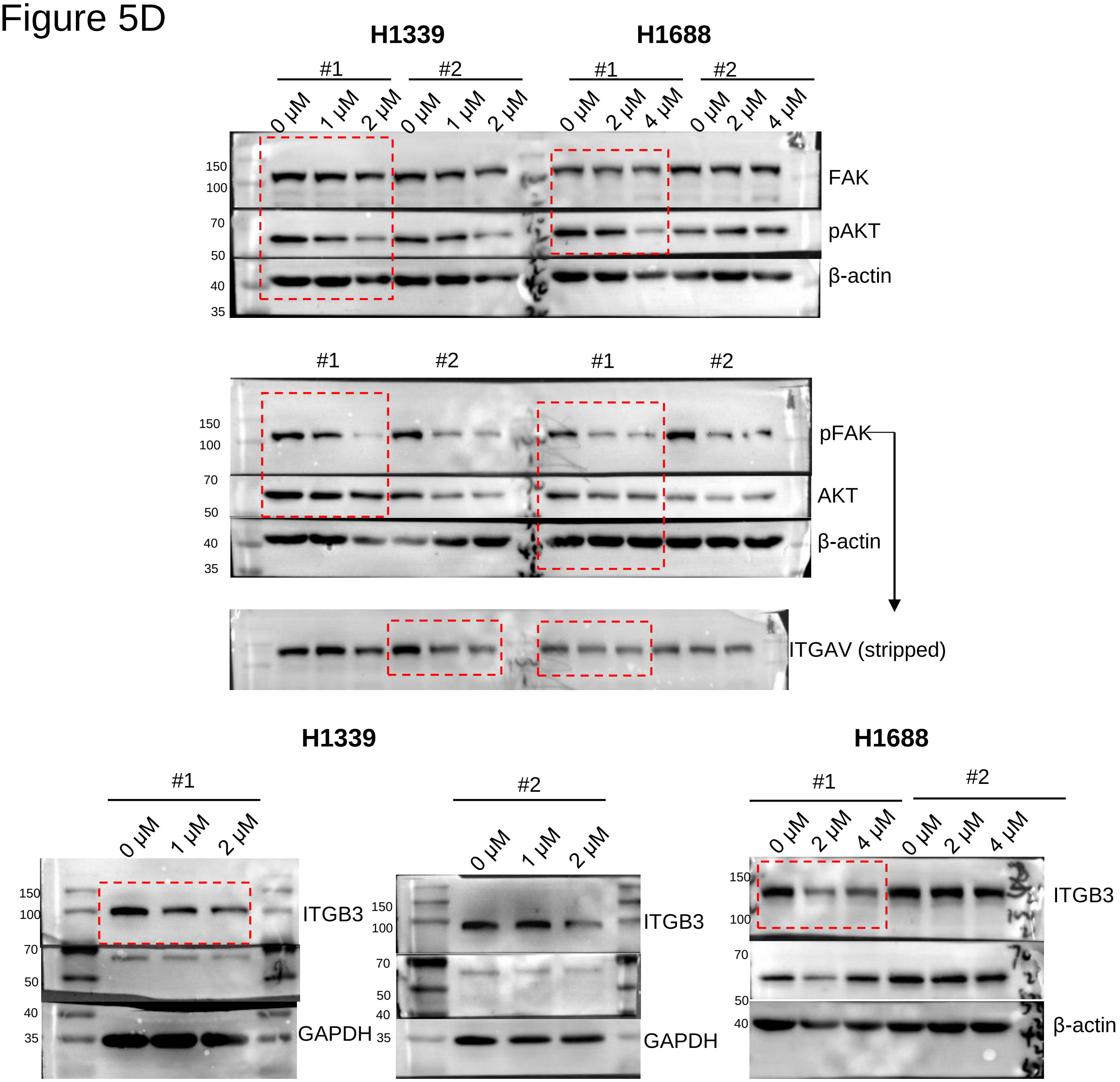
H1339
H1688
#1
#2
#1
#2
0 µM 1 µM 2 µM 0 µM 1 µM 2 µM
0 µM 2 µM 4 µM 0 µM 2 µM 4 µM
150
100
70
50
40
35
FAK
pAKT
β-actin
#1
#2
#1
#2
150
100
70
50
40
35
pFAK
AKT
β-actin
ITGAV (stripped)
H1339
H1688
#1
0 µM 1 µM 2 µM
150
100
70
50
40
35
ITGB3
GAPDH
#2
0 µM 1 µM 2 µM
150
100
70
50
40
35
ITGB3
GAPDH
#1
#2
0 µM 2 µM 4 µM 0 µM 2 µM 4 µM
150
100
70
50
40
ITGB3
β-actin

Figure 5F

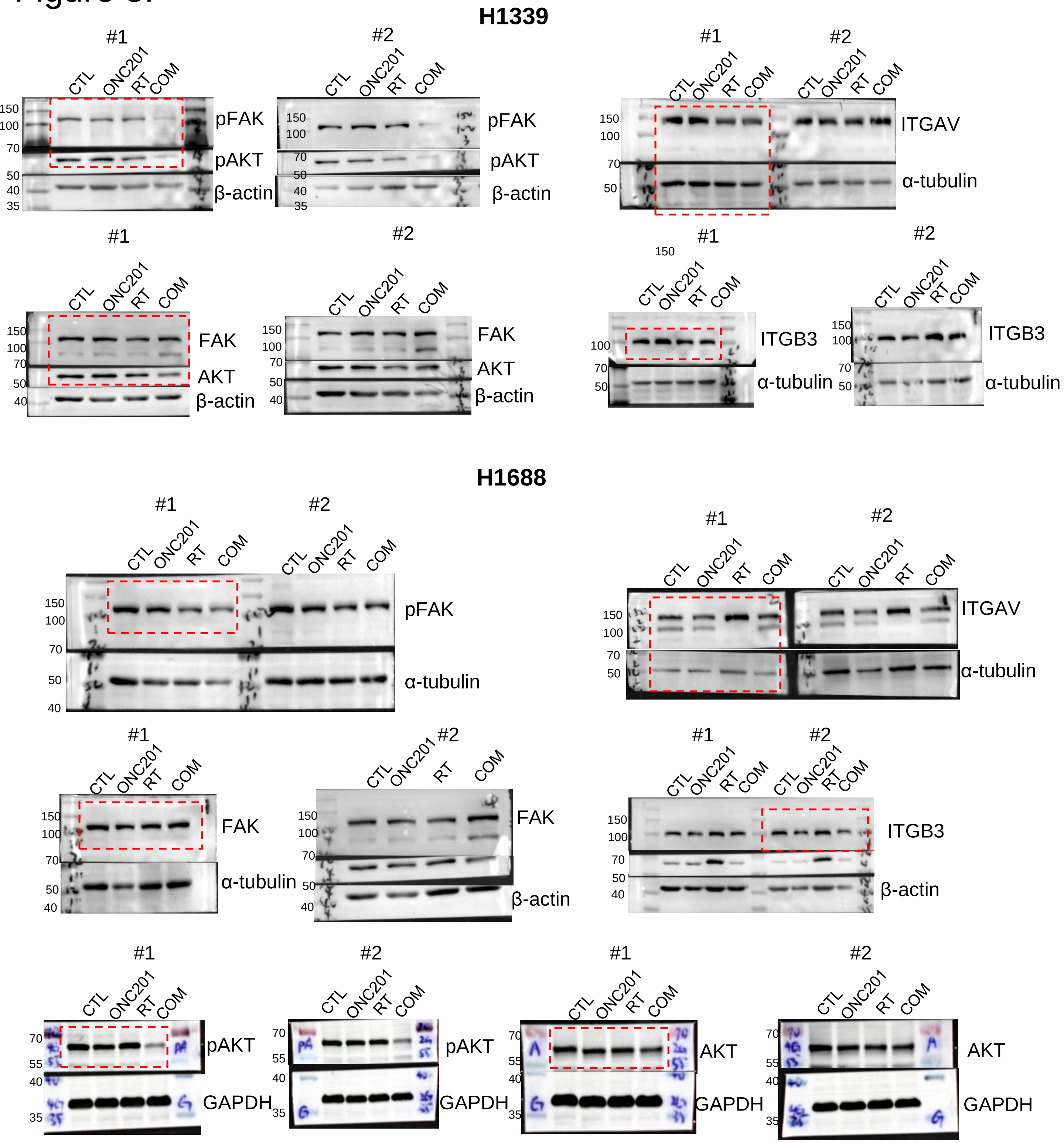
H1339
#1
#2
CTL
ONC201
RT
COM
pFAK
pAKT
β-actin
ITGAV
α-tubulin
FAK
AKT
ITGB3
150
100
70
50
40
35
H1688
GAPDH
55

Figure 6A

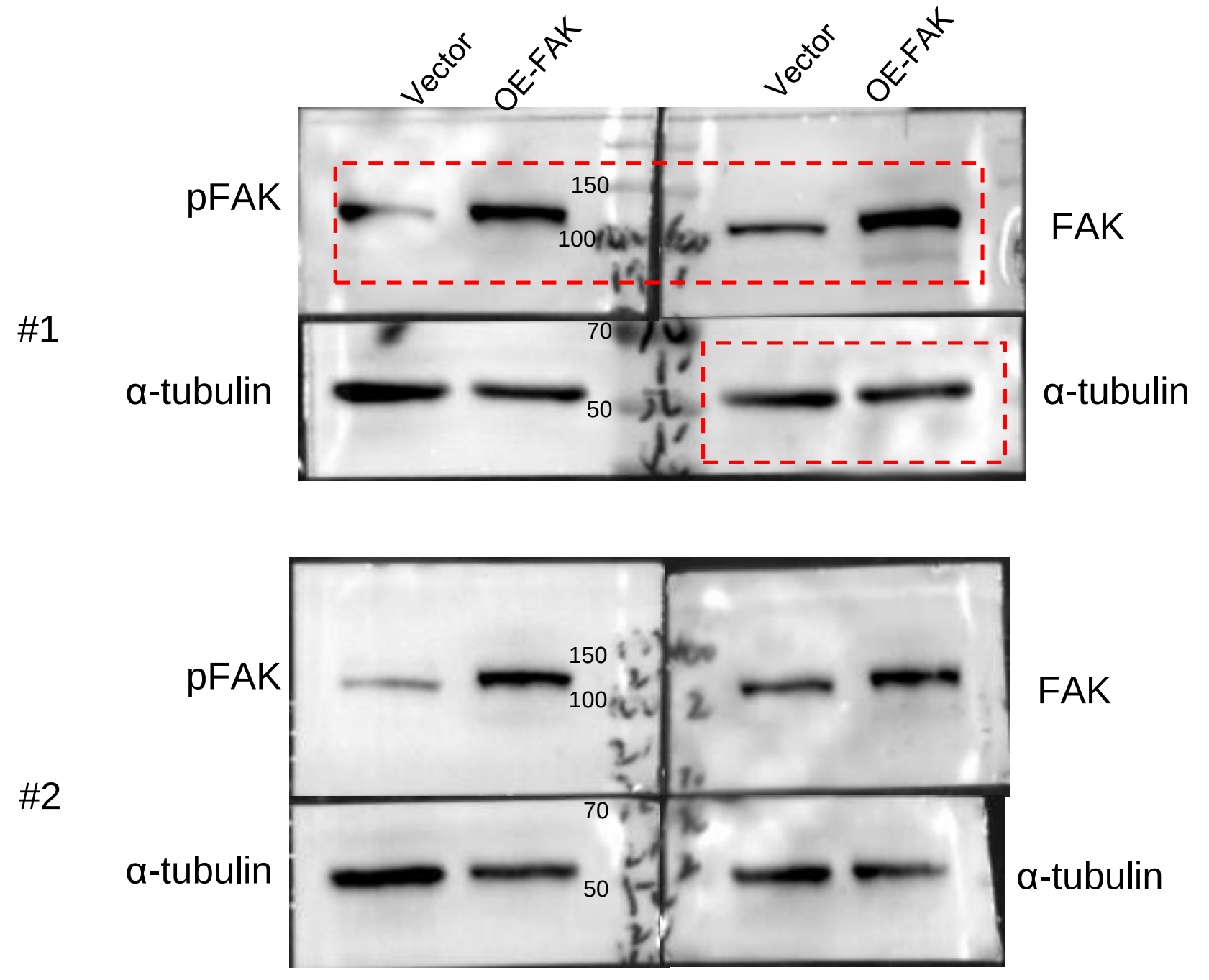

Figure 6E

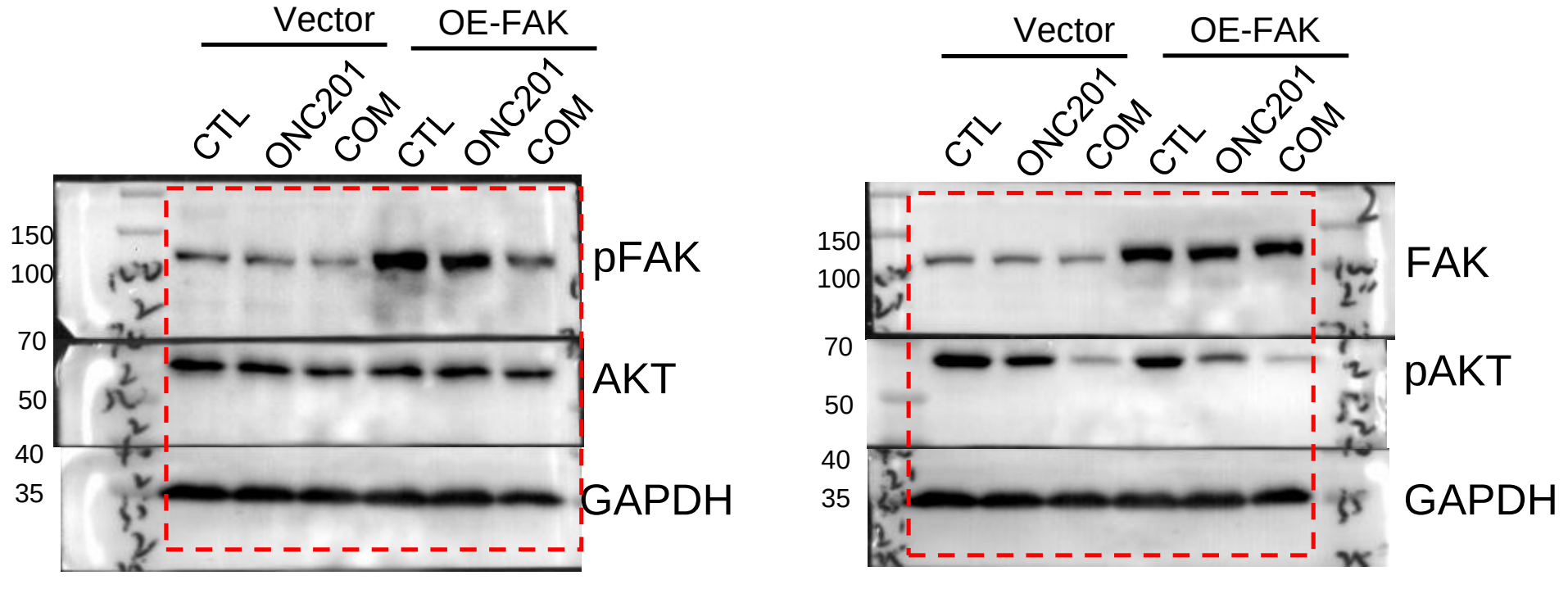

Figure S4A

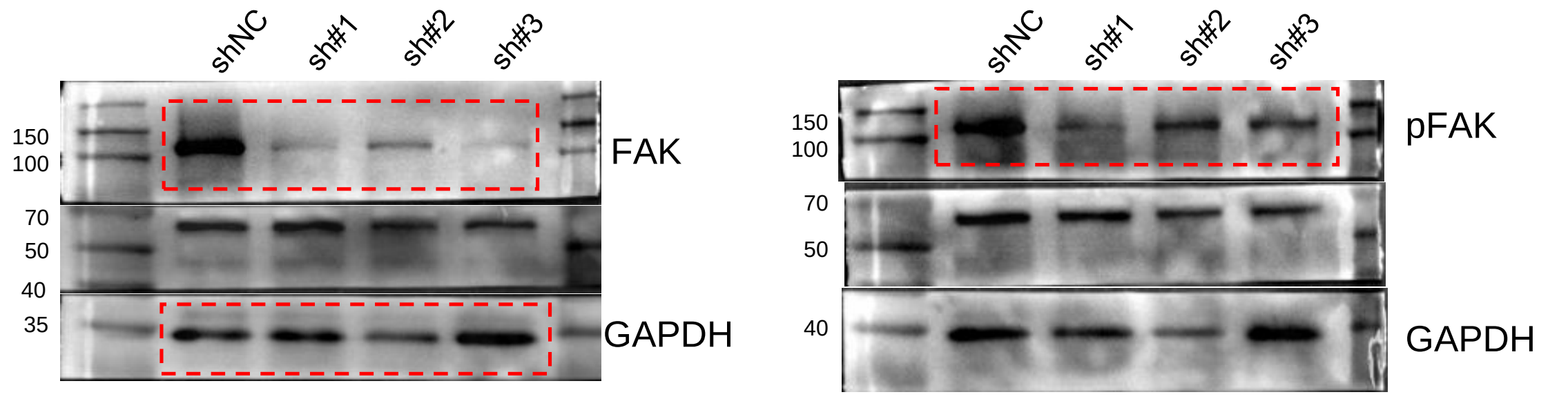

Figure S4E

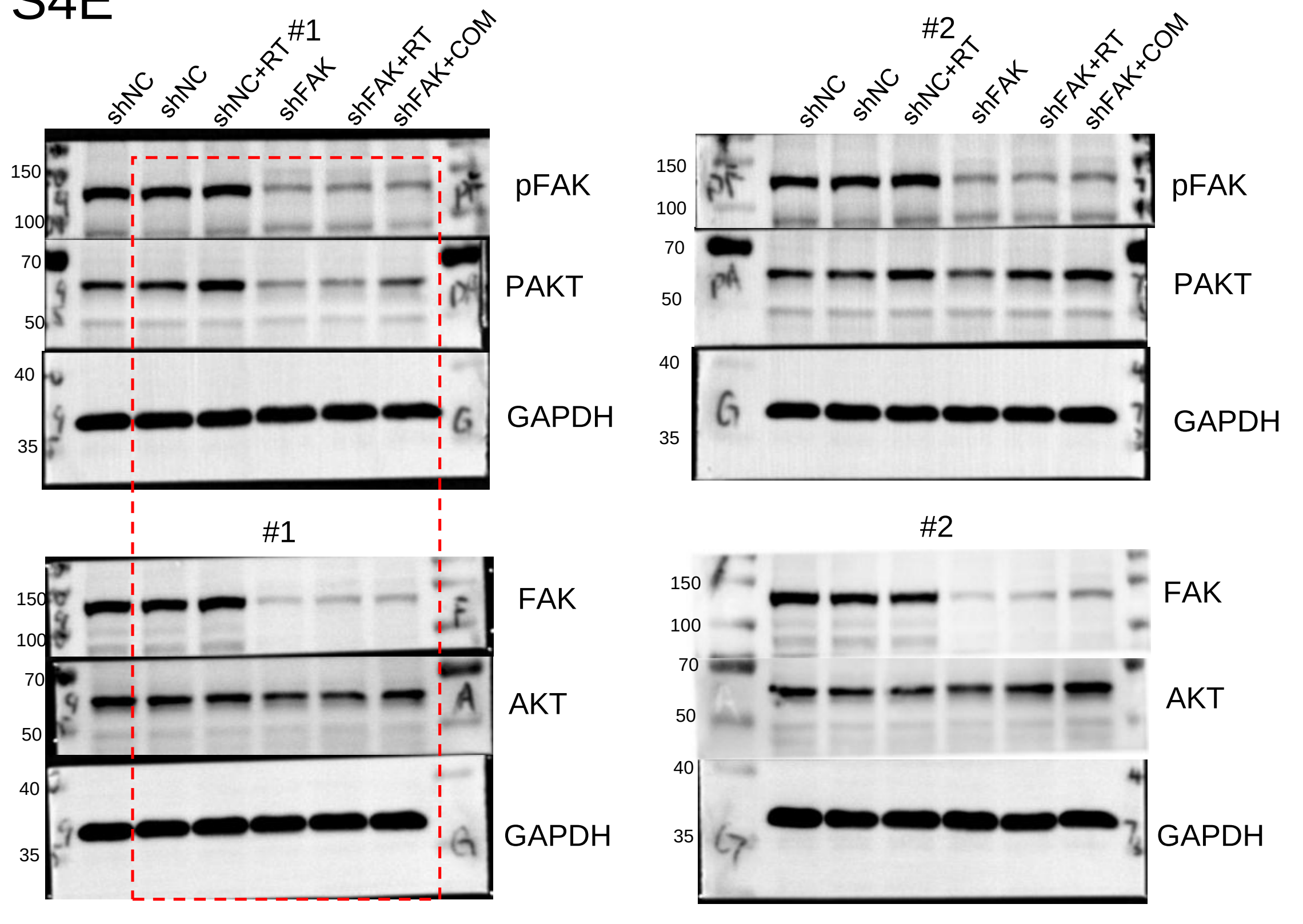

Data S2：Unedited microscopy images Figure 1K, N

Figure 1K

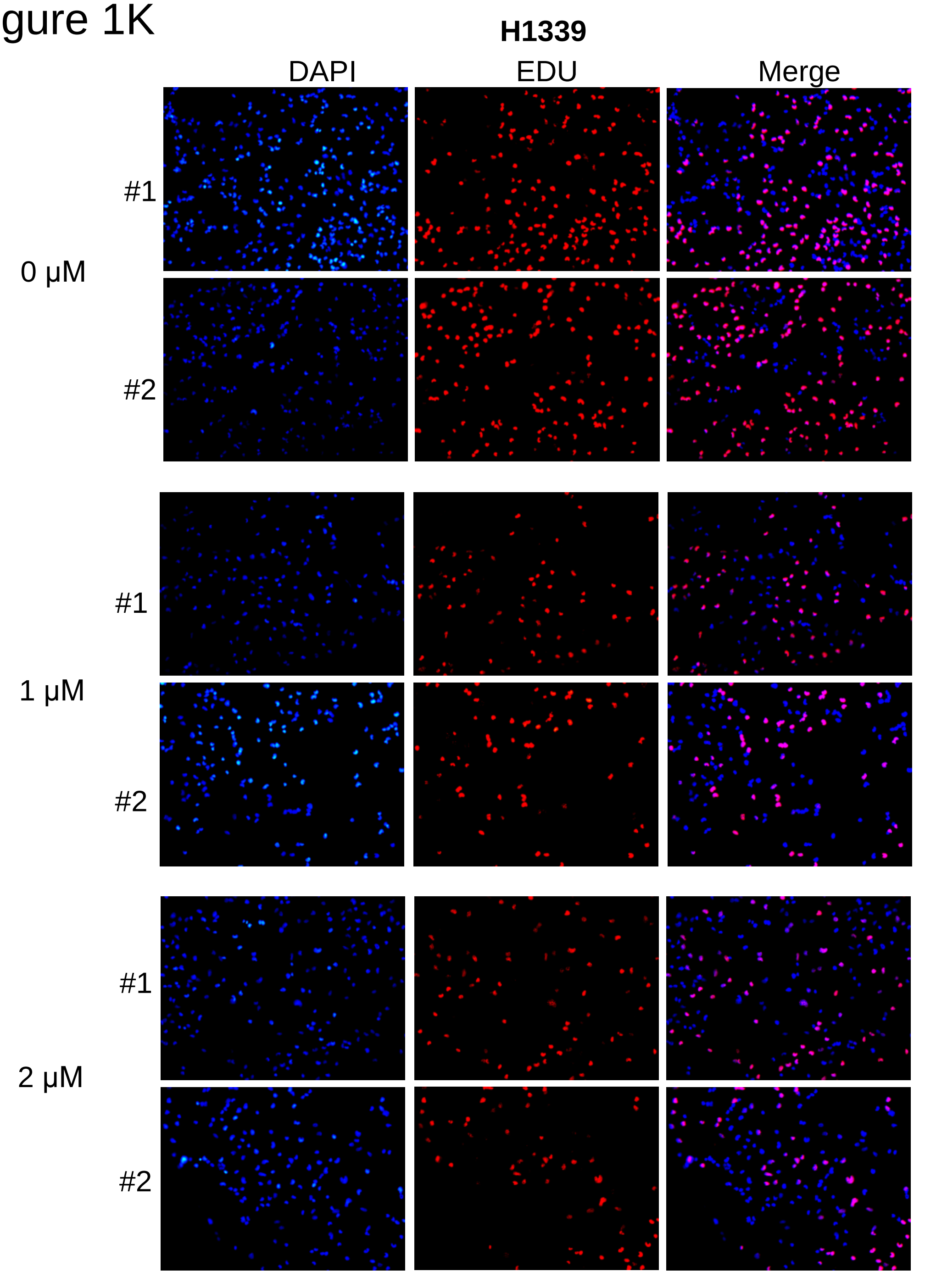

Figure 1K

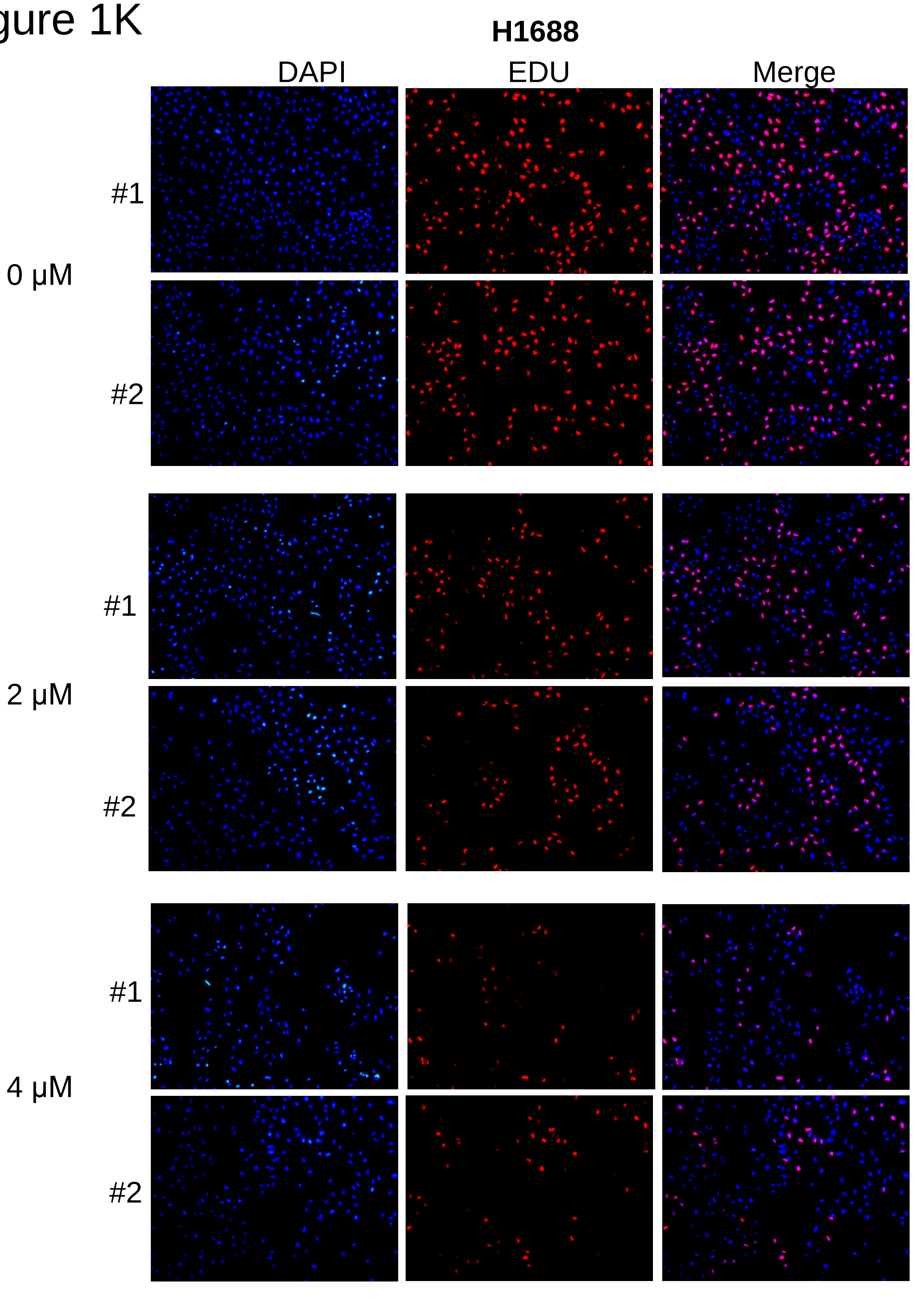

Figure 1N

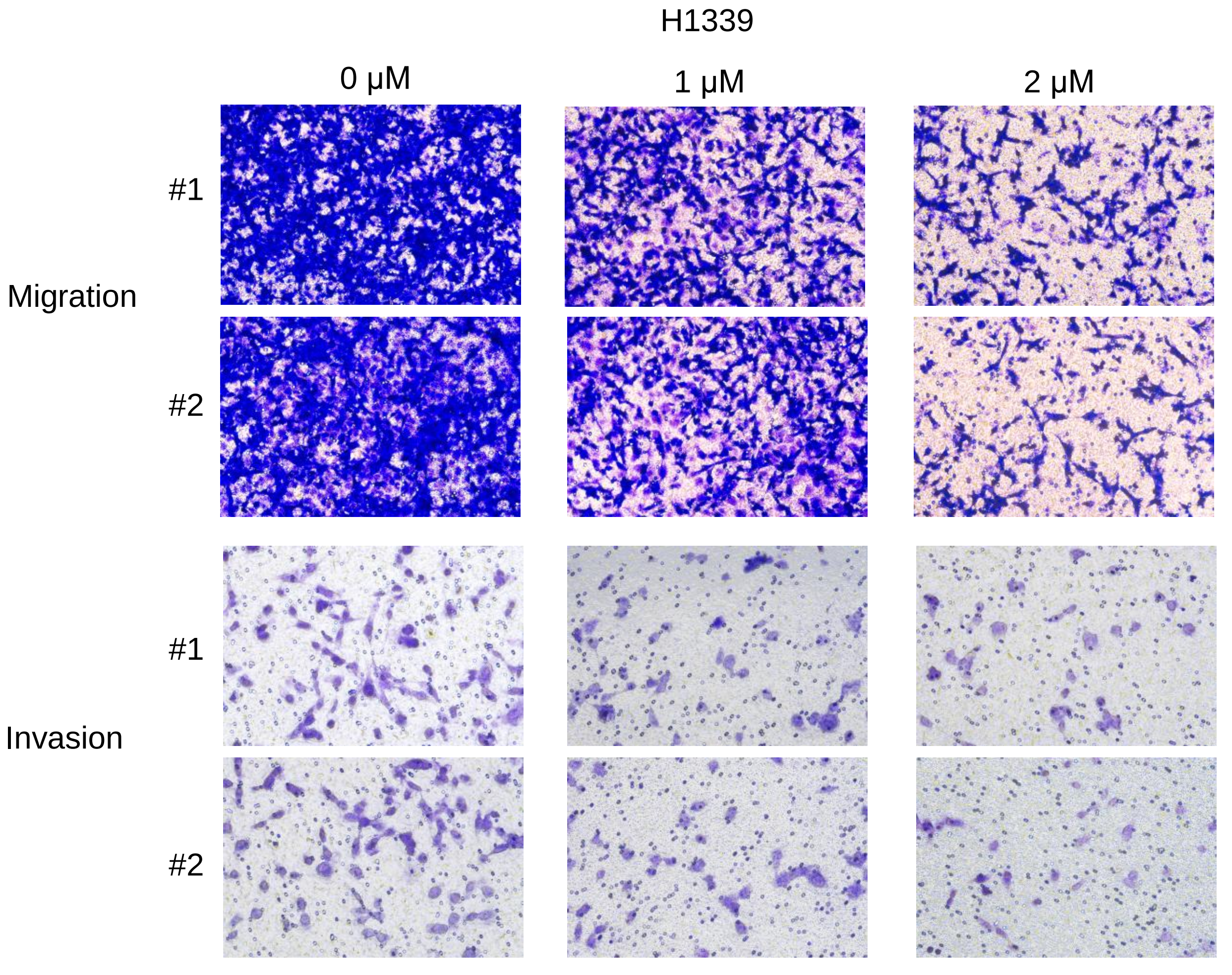

Figure 1N

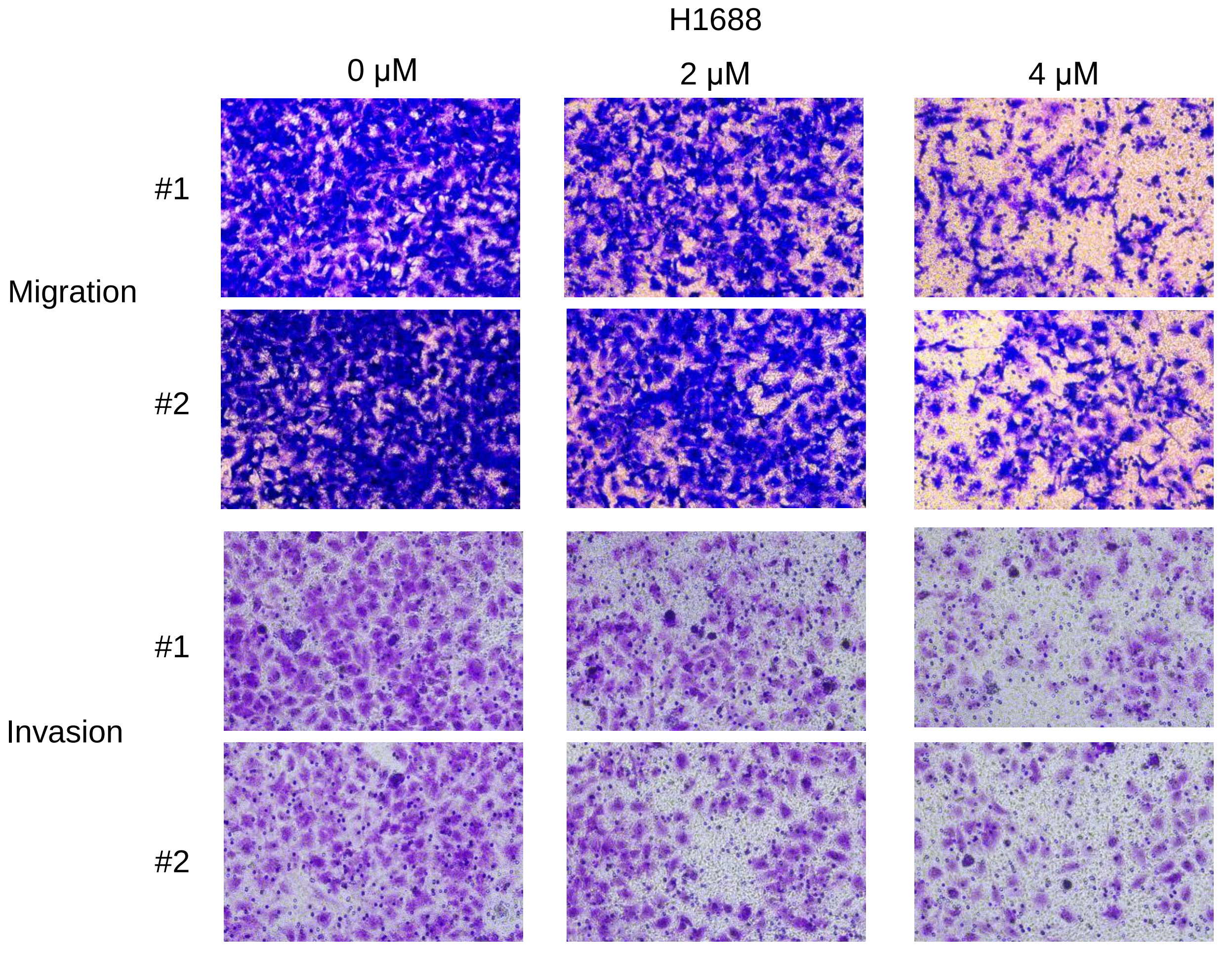

Data S3: Unedited microscopy images Figure 7D, S5D

Figure 7D

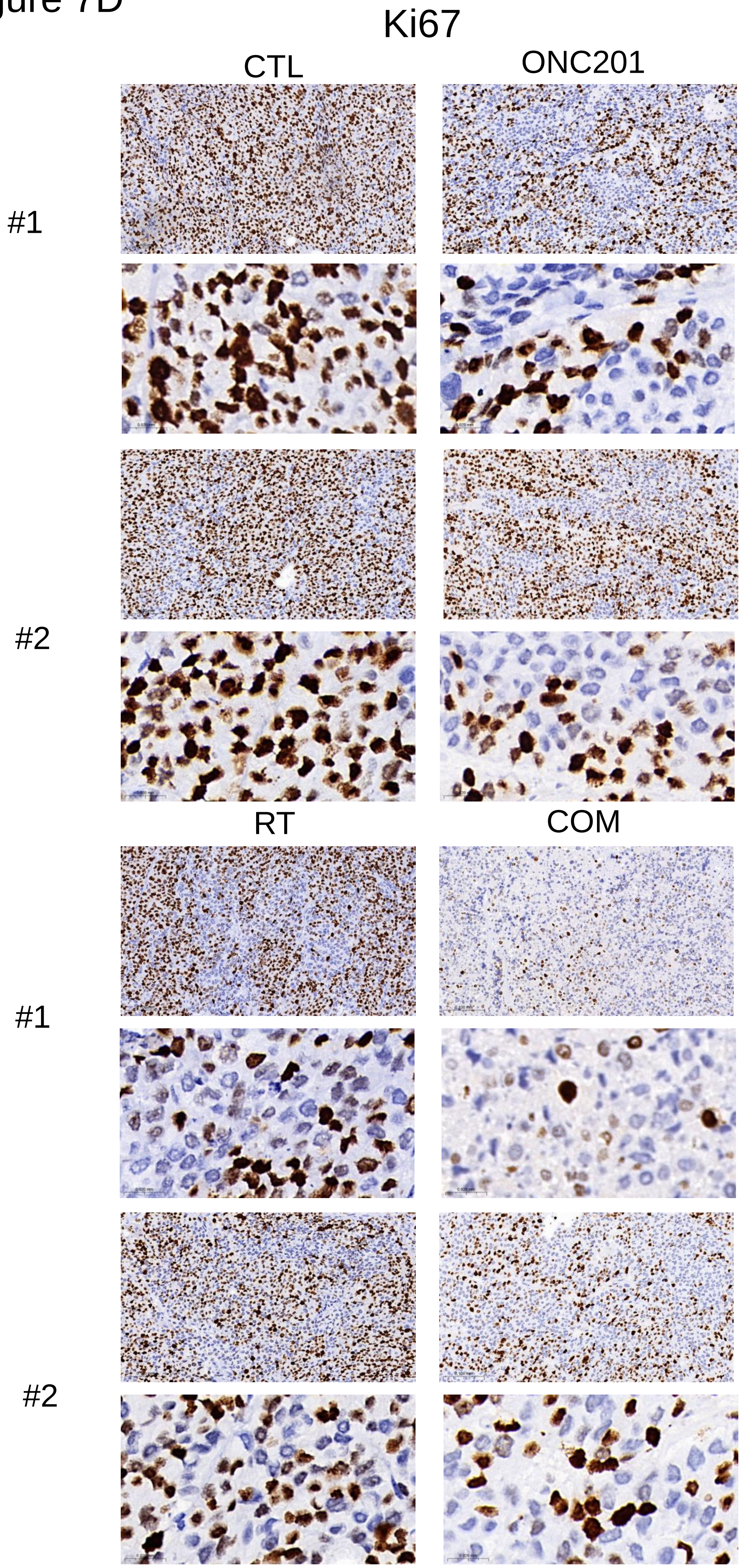

Figure 7D

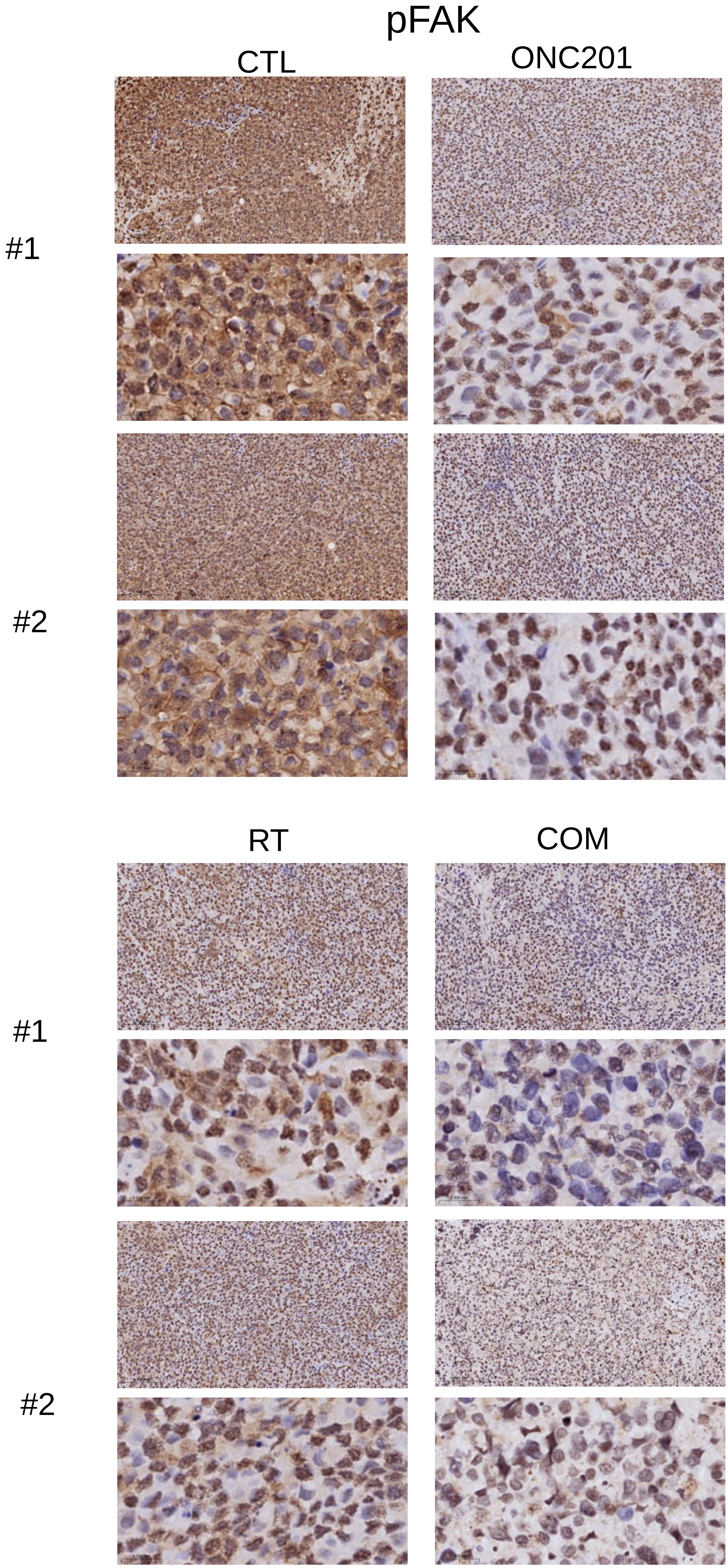

Figure 7D

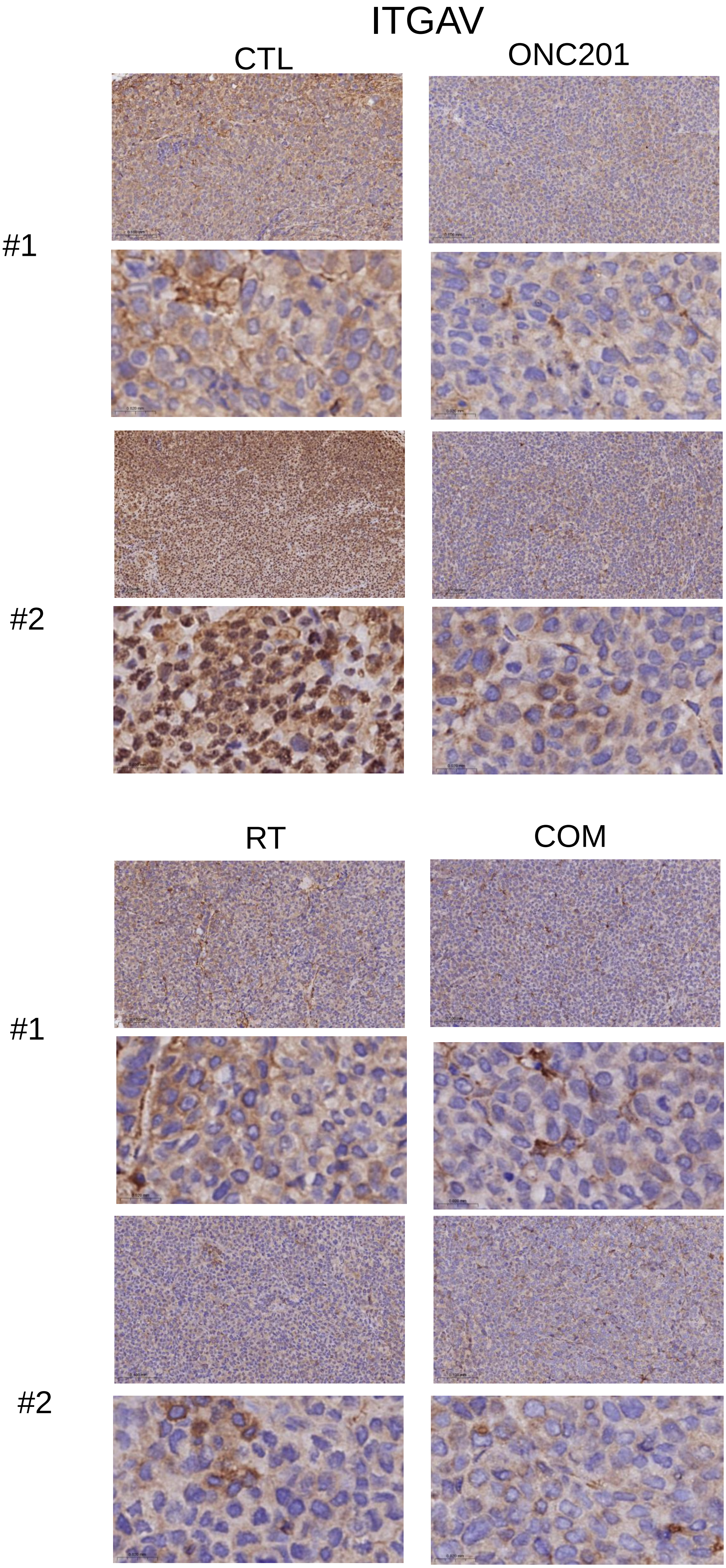

Figure 7D

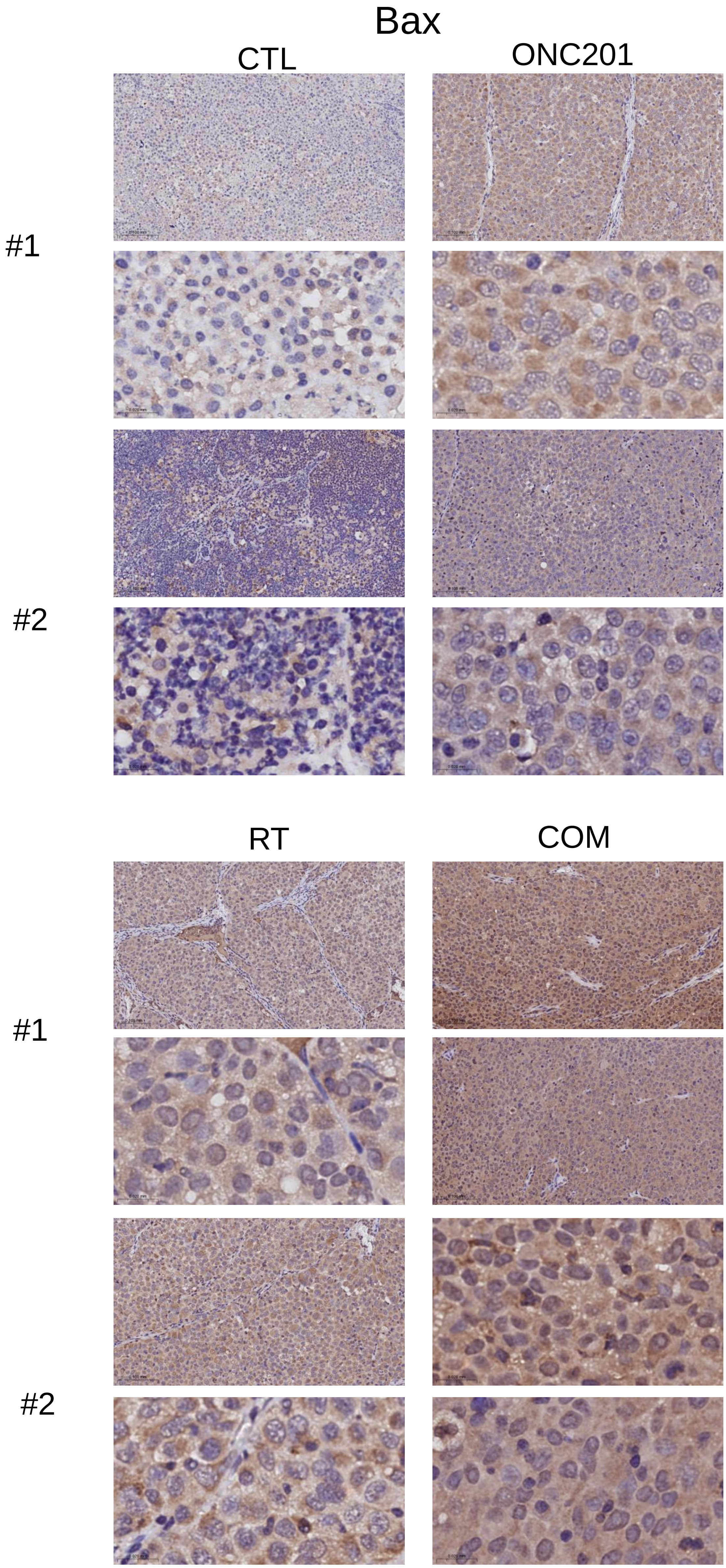

Figure 7D

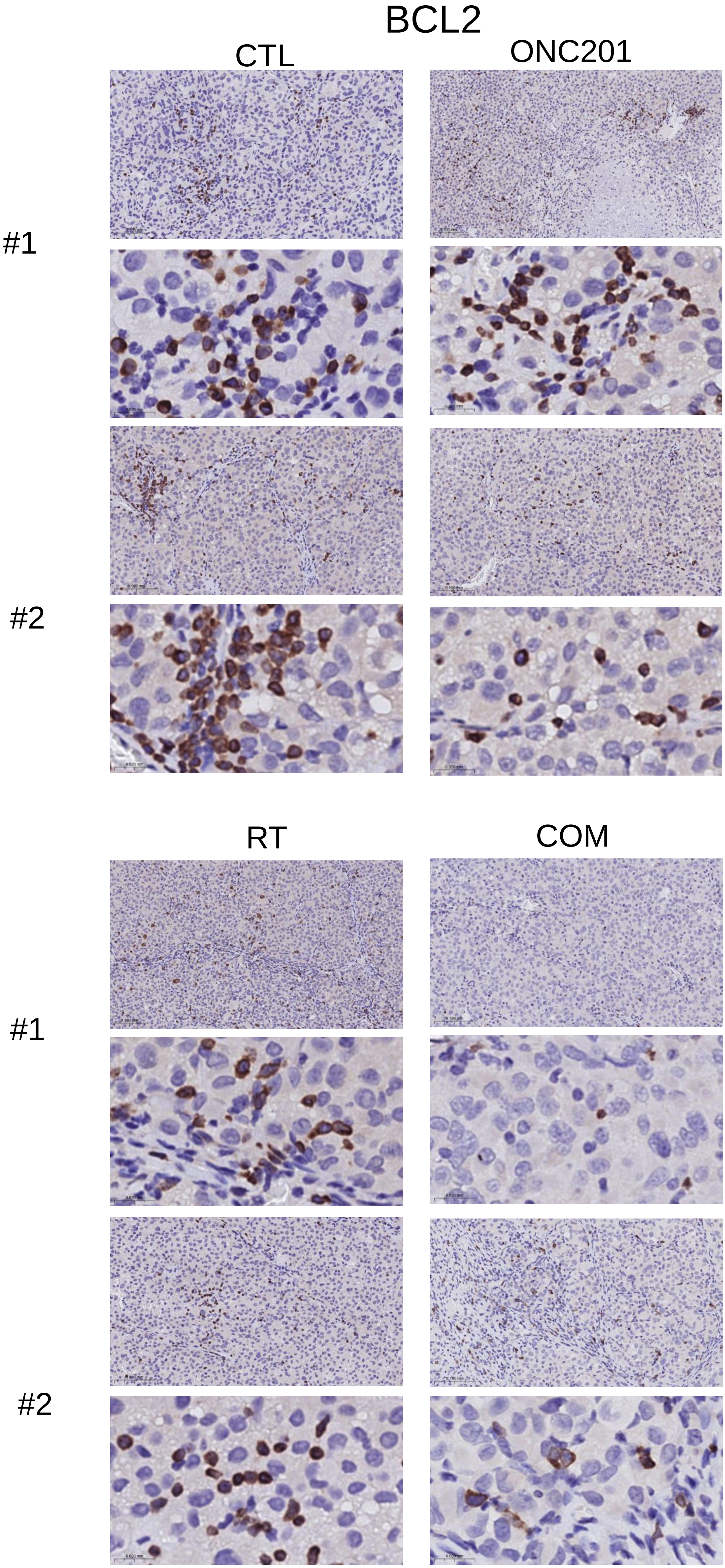

Figure S5D

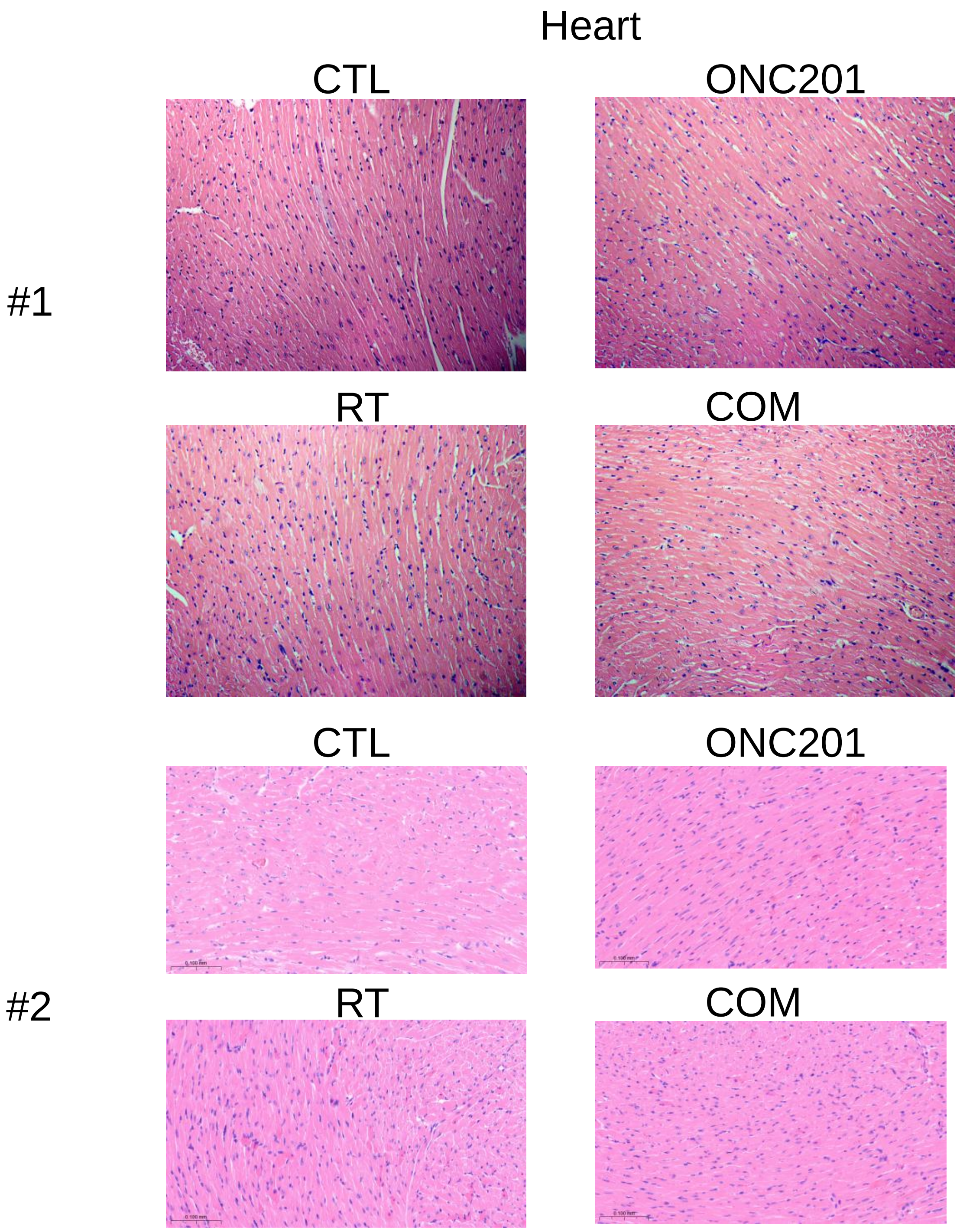

Figure S5D

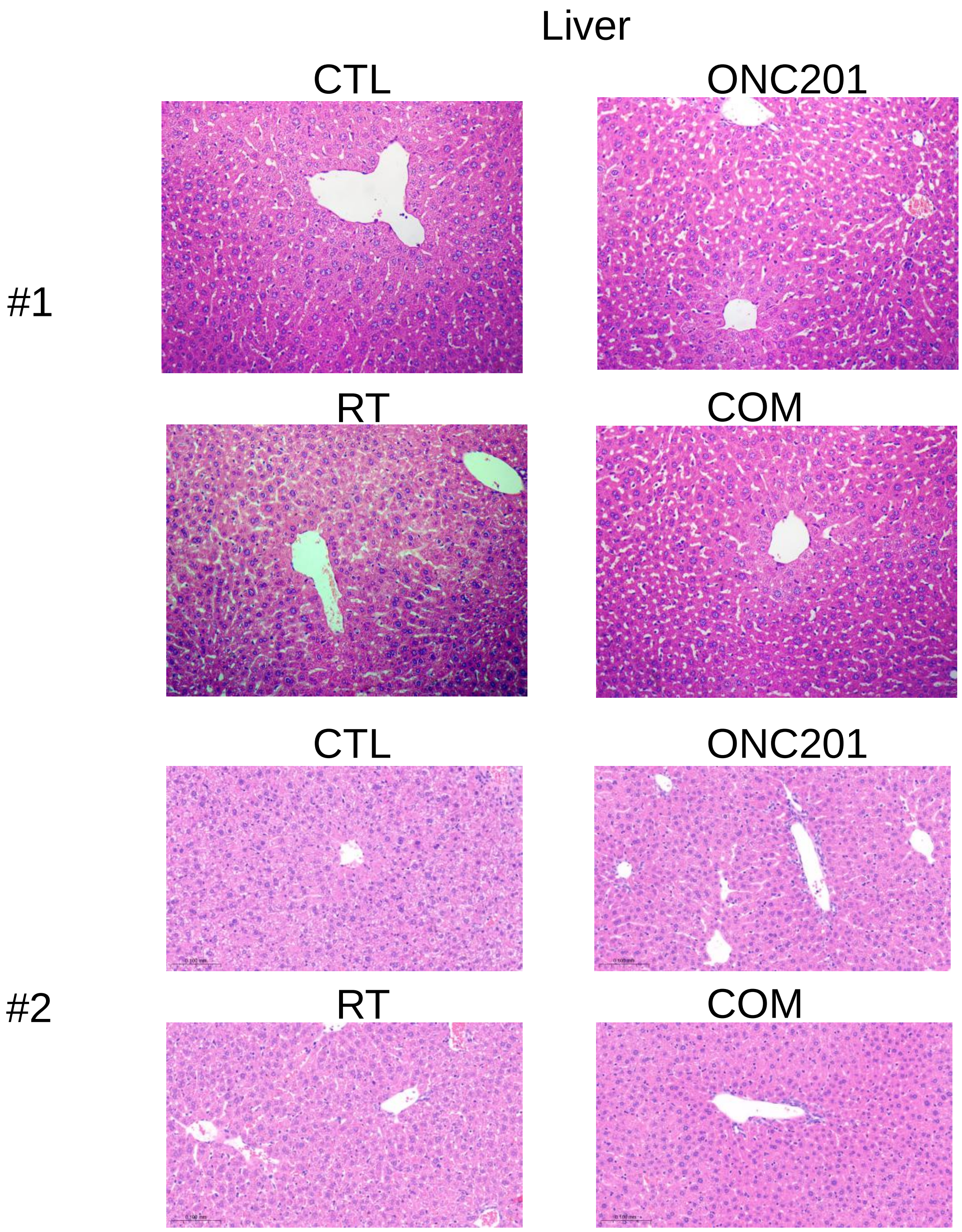

Figure S5D

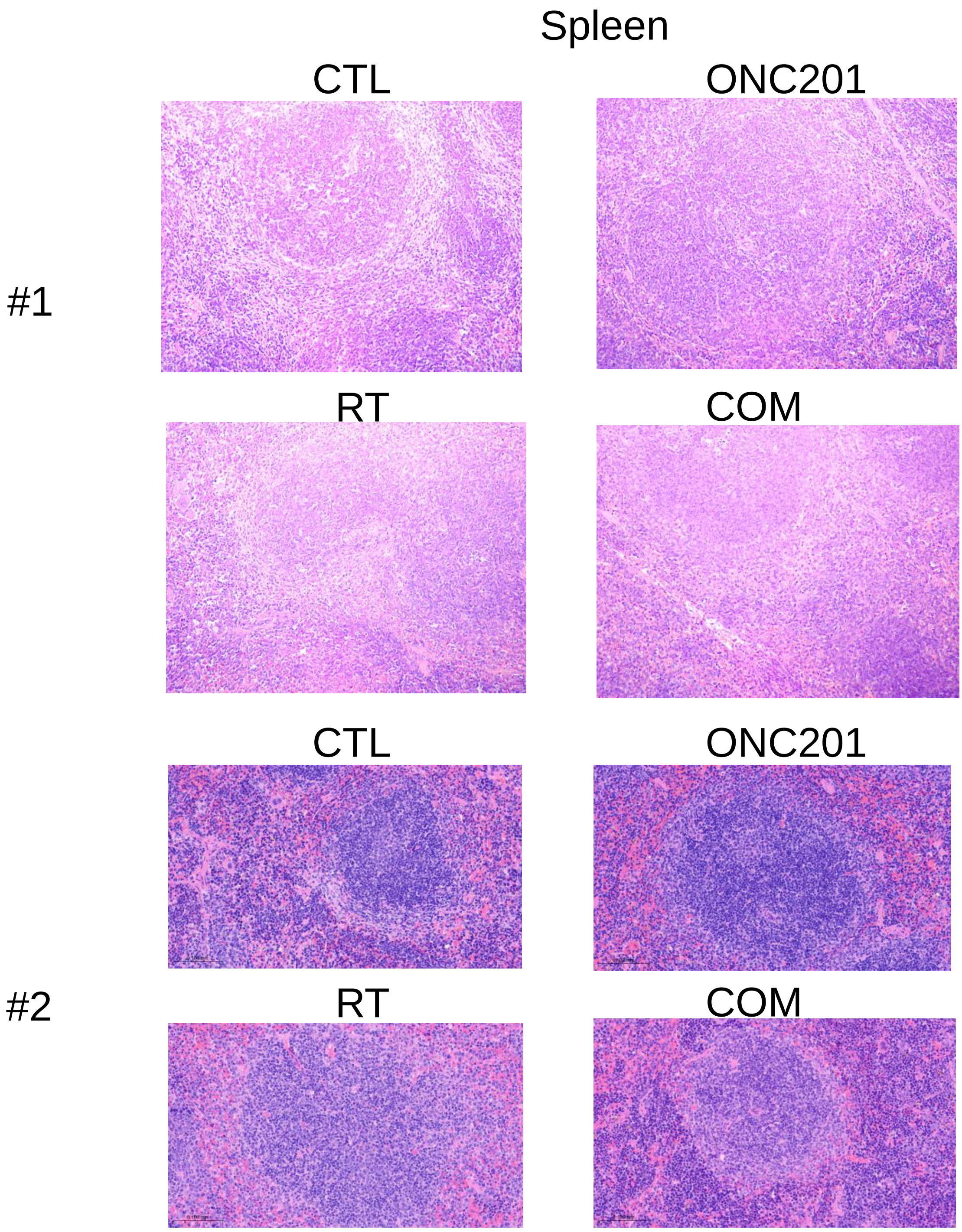

Figure S5D

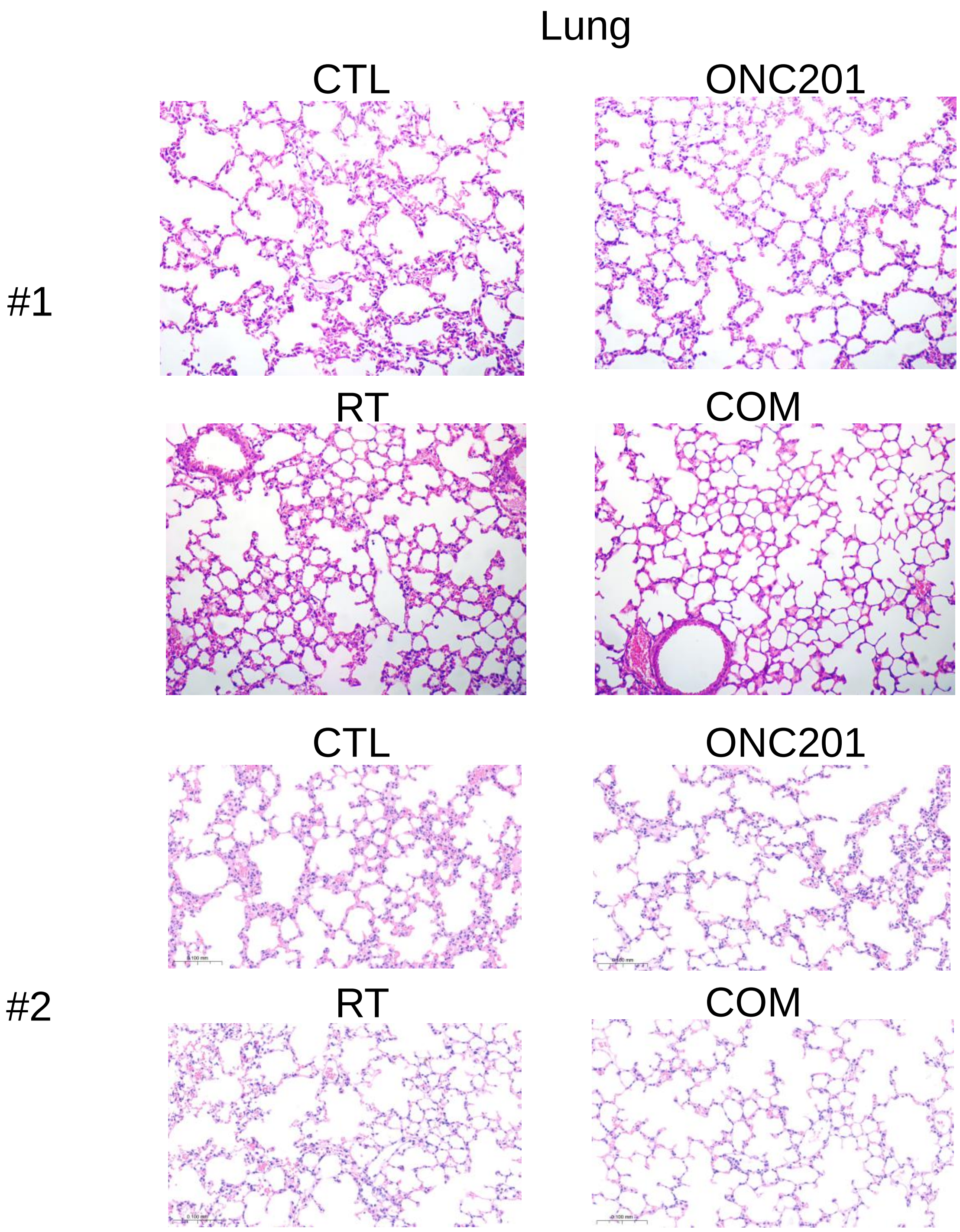

Figure S5D

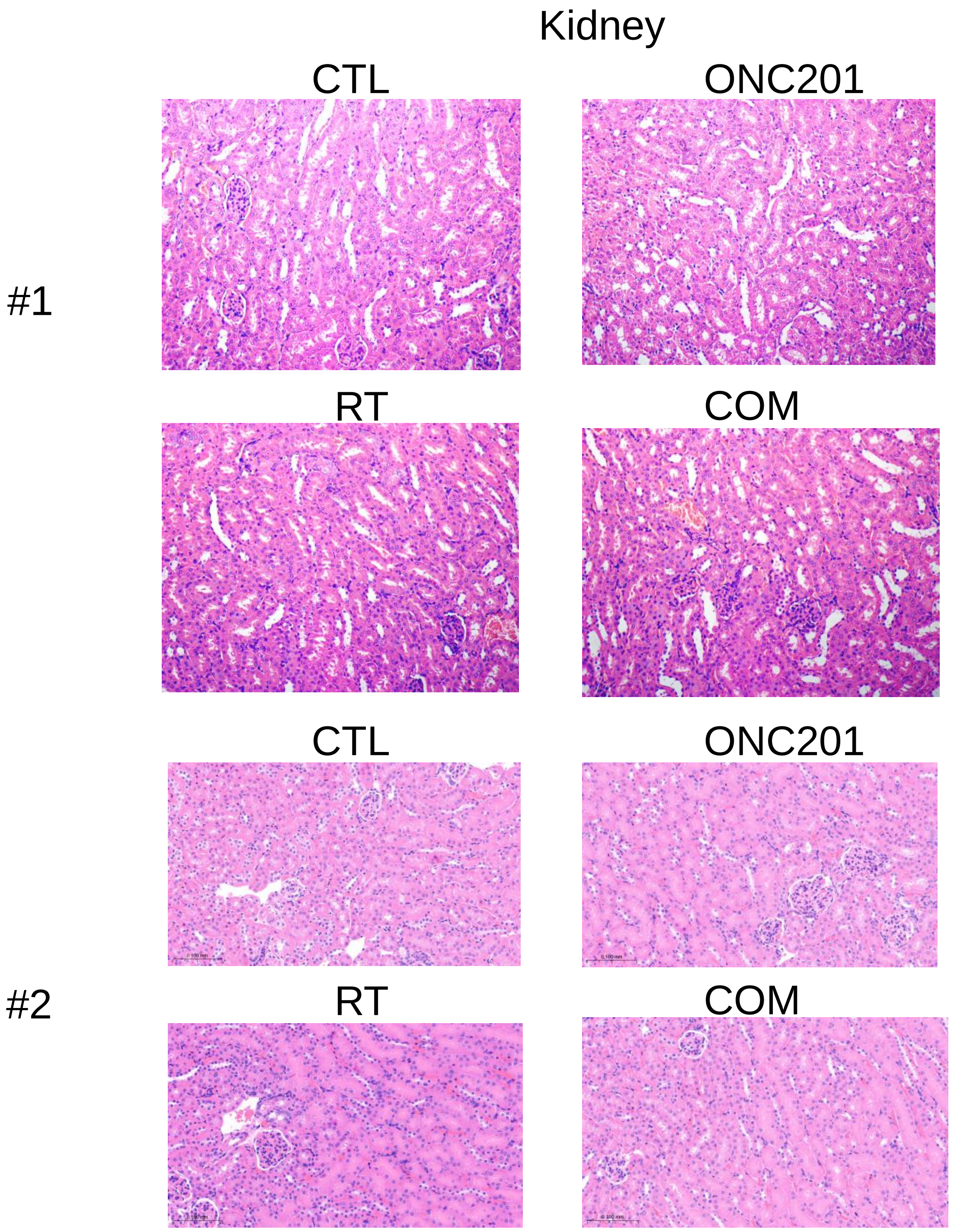

Data S4 : Unedited microscopy images Figure S2A-B

Figure S2A

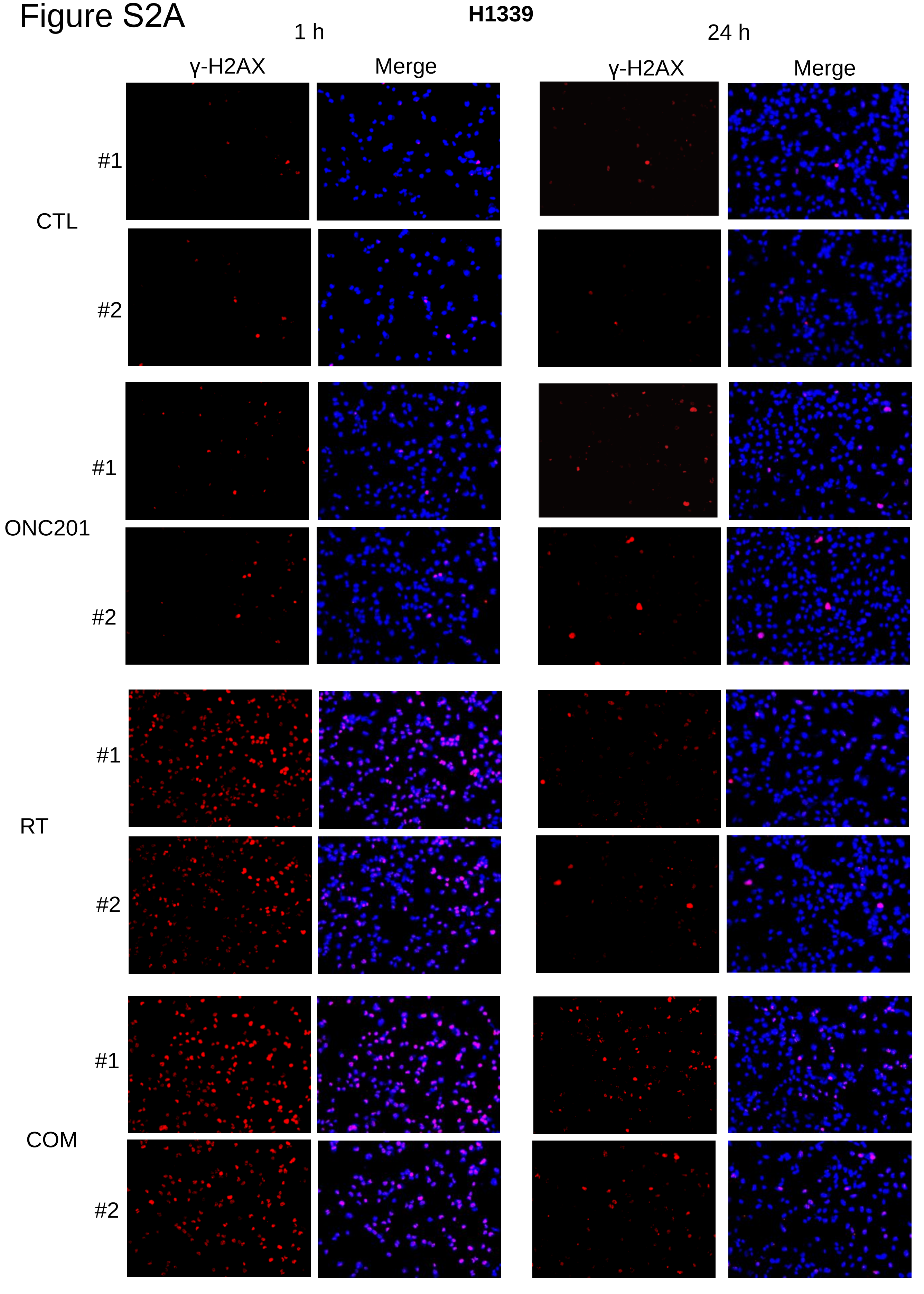

Figure S2B

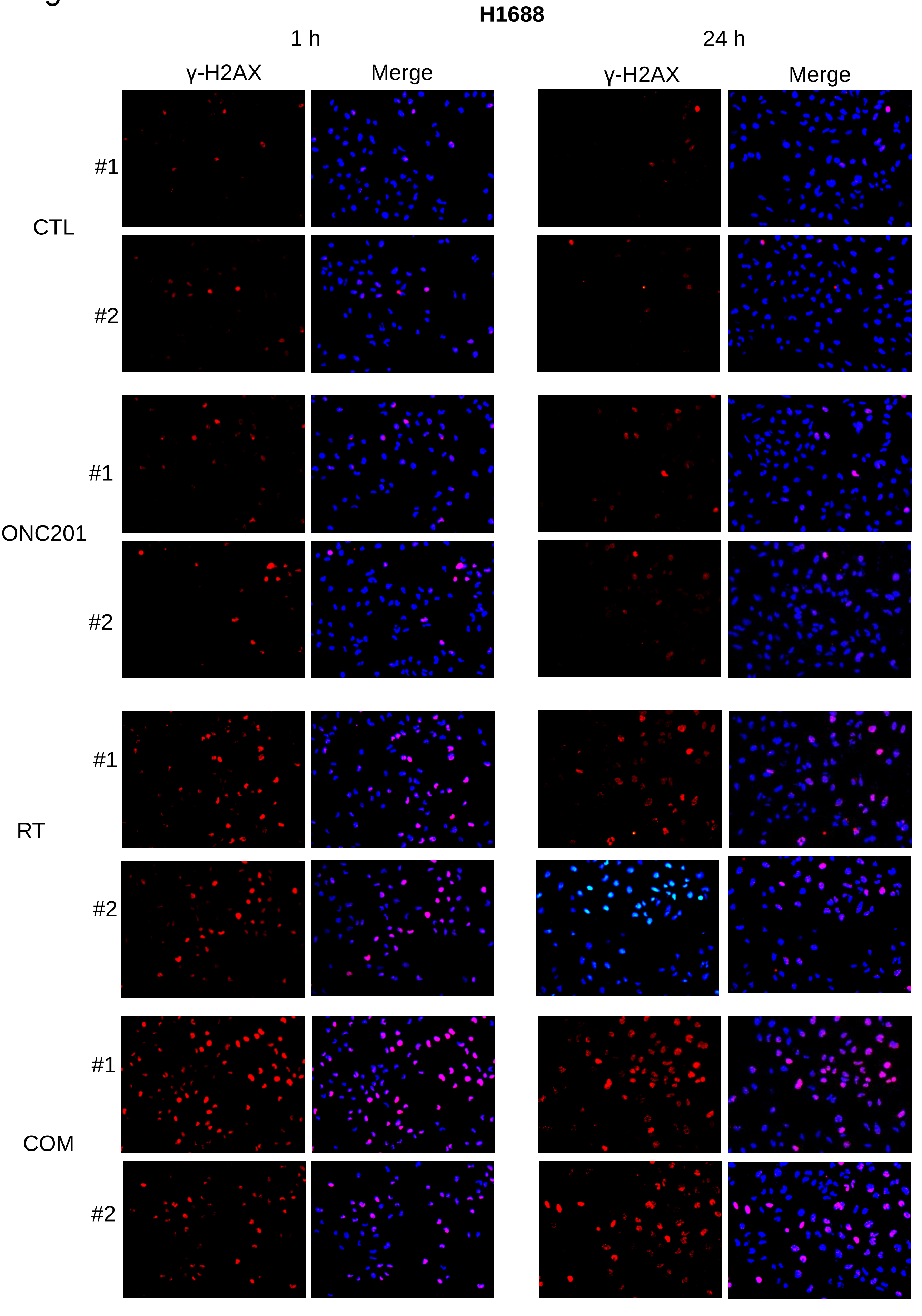
H1688
1 h
24 h
γ-H2AX
Merge
γ-H2AX
Merge
CTL
#1
#2
ONC201
#1
#2
RT
#1
#2
COM
#1
#2
